# Genetic regulation of body size and morphology in children: a twin study of 22 anthropometric traits

**DOI:** 10.1038/s41366-023-01253-0

**Published:** 2023-01-12

**Authors:** Karri Silventoinen, José Maia, Weilong Li, Reijo Sund, Élvio R. Gouveia, António Antunes, Gonçalo Marques, Martine Thomis, Aline Jelenkovic, Jaakko Kaprio, Duarte Freitas

**Affiliations:** 1 Population Research Unit, Faculty of Social Sciences, University of Helsinki, Helsinki, Finland; 2 Center of Research, Education, Innovation and Intervention in Sport (CIFI2D), Faculty of Sport, University of Porto, Porto, Portugal; 3 Institute of Clinical Medicine, University of Eastern Finland, Kuopio, Finland; 4 Department of Physical Education and Sport, University of Madeira, Funchal, Portugal; 5 LARSYS, Interactive Technologies Institute, Funchal, Portugal; 6 Physical Activity, Sports & Health Research Group, Department of Movement Sciences, Faculty of Movement and Rehabilitation Sciences, KU Leuven, Leuven, Belgium; 7 Department of Genetics, Physical Anthropology and Animal Physiology, Faculty of Science and Technology, University of the Basque Country, Bilbao, Spain; 8 Institute for Molecular Medicine Finland (FIMM), HiLIFE, University of Helsinki, Helsinki, Finland

**Keywords:** Anatomy, Genetics

## Abstract

**Background:**

Anthropometric measures show high heritability, and genetic correlations have been found between obesity-related traits. However, we lack a comprehensive analysis of the genetic background of human body morphology using detailed anthropometric measures.

**Methods:**

Height, weight, 7 skinfold thicknesses, 7 body circumferences and 4 body diameters (skeletal breaths) were measured in 214 pairs of twin children aged 3–18 years (87 monozygotic pairs) in the Autonomous Region of Madeira, Portugal. Factor analysis (Varimax rotation) was used to analyze the underlying structure of body physique. Genetic twin modeling was used to estimate genetic and environmental contributions to the variation and co-variation of the anthropometric traits.

**Results:**

Together, two factors explained 80% of the variation of all 22 anthropometric traits in boys and 73% in girls. Obesity measures (body mass index, skinfold thickness measures, as well as waist and hip circumferences) and limb circumferences loaded most strongly on the first factor, whereas height and body diameters loaded especially on the second factor. These factors as well as all anthropometric measures showed high heritability (80% or more for most of the traits), whereas the rest of the variation was explained by environmental factors not shared by co-twins. Obesity measures showed high genetic correlations (0.75–0.98). Height showed the highest genetic correlations with body diameter measures (0.58–0.76). Correlations between environmental factors not shared by co-twins were weaker than the genetic correlations but still substantial. The correlation patterns were roughly similar in boys and girls.

**Conclusions:**

Our results show high genetic correlations underlying the human body physique, suggesting that there are sets of genes widely affecting anthropometric traits. Better knowledge of these genetic variants can help to understand the development of obesity and other features of the human physique.

## Introduction

Anthropometric measures are the key method to assess a child’s nutrition and development [1]. While body mass index (BMI), waist circumference and skinfold thicknesses are important to measure excess energy intake [2], height [3], upper arm circumference [4] and chest circumference [5] provide important information on malnutrition. Genetic studies of anthropometric traits are important for understanding the factors behind physical development and can thus also provide new insight into the role of environmental factors. The genetics of height and BMI have been extensively studied in children using the classic twin design [6, 7]. Further, molecular genetic studies using mainly the genome-wide-association (GWA) design [8, 9] have identified thousands of loci affecting adult height and BMI which show strong genetic correlations with these traits over childhood and adolescence [10]. There are also genetic twin studies on other traits, such as waist circumference [11], skinfold thicknesses [12], and chest circumference [13], as well as head circumference and several other craniofacial measures [14]. Still, generally, less is known about the genetics of anthropometric traits other than height and BMI. Collectively, these studies highlight the importance of genetic factors behind the variation of anthropometric traits.

However, an area which is still poorly understood is how much these different anthropometric measures share common genetic variation. Previous twin studies have shown genetic correlations between BMI and waist circumference [11], as well as BMI and several skinfold thicknesses [15, 16]. Genetic correlations were also found in a family-pedigree study including detailed obesity and other anthropometric measures [17]. These results based on twin and family designs have been confirmed by a GWA study finding genetic correlations between childhood BMI and percentage of body fat as well as waist and hip circumferences in adulthood [18]. There can also be shared genetic background even between distinct body traits as demonstrated in a family-pedigree study finding genetic correlations between craniofacial traits and body composition [19]. These genetic correlations can reflect genetic pleiotropy going back to fetal development [20]. Further, genetic factors can affect the adipose tissue both directly [21] and indirectly through, for example, eating behavior [22], thus creating correlations between indicators of obesity.

Knowledge on the genetic correlations between anthropometric traits can provide insight into the genetic regulation and development of body morphology. This knowledge may also have practical implications since it can guide which traits are most informative to assess obesity. However, a limitation in the previous studies is that they include only a few traits and thus can only partly capture the complexity of the human body physique. In this study, we use a twin data set of children that includes 22 anthropometric measures providing detailed information on human body size and morphology. Using genetic twin modeling, we analyze how these traits are mutually correlated and how much they share common genetic variation.

## Data and methods

The data were derived from the Madeira Twin Study conducted in the Autonomous Region of Madeira, Portugal [23]. First, all public and private schools were contacted and asked if they had twins as students and inquired about their contact information. Together, 434 twin families were identified, and an invitation letter to participate in the study was sent to them. From these families, 216 families having twin children 3 to 18 years of age (51% girls) participated in a detailed clinical examination in the capital city of Funchal in 2007 and 2008. During the examination, the children gave a blood sample. Zygosity was assessed by the polymerase chain reaction (PCR) amplification of short tandem repeat analyzed with a commercially available panel (AmpFlSTR Identifiler kit) comprising 15 autosomal, codominant, unlinked loci and the sex-determining marker [24]. Among the twin pairs, 87 were monozygotic (MZ), 73 same-sex dizygotic (SSDZ) and 56 opposite-sex dizygotic (OSDZ) pairs. The twins themselves and/or their parents/legal guardians provided written informed consent. The Scientific Board of the University of Madeira approved the study protocol.

A team of six experienced researchers from the Laboratory of Growth and Development of the University of Madeira conducted detailed anthropometric measures based on a standardized protocol [25]. All measures were done in a swimsuit, without shoes and with jewelry removed. All one-sided measurements were taken on the left side of the body. Height was measured using a Harpenden wall-mounted stadiometer accurate to 1 mm (Holtain, UK). Body weight was measured on a balance-beam scale accurate to 0.1 kg (Scena Optima 760, UK). BMI was then calculated by dividing weight in kg by the square of height in meters (kg/m^2^). Four body diameters (biacromial, bicristal, humerus and femur) were measured with a spreading caliper with an accuracy of 1 mm (Siber-Hegner, GPM, Switzerland). Seven body circumferences (waist, hip, calf, thigh, upper arm, forearm and upper arm flexed) were measured with a flexible steel tape accurate to 1 mm (Holtain, UK). Seven skinfold thicknesses (triceps, biceps, subscapular, suprailiac, calf, front thigh and abdominal) were assessed using a skinfold caliper and recorded to the nearest 2 mm (Siber-Hegner, GPM, Switzerland). We did not have missing cases in our data. However, we removed a few outliers (1 measurement for femur diameter and calf circumference and 2 measurements for humerus diameter, as well as thigh and forearm circumferences) since otherwise they may have disproportionally affected heritability estimates.

We found that the distributions of waist and hip circumferences, BMI, weight and all seven skinfold thicknesses were skewed and thus used logarithmic transformation to normalize them. After this transformation, the distributions of all traits were roughly normal (the skewness parameters varied between 0.06 and 0.98). In our previous study reporting the heritability estimates of 10 of these 22 traits, we did not find systematic differences when comparing children younger and older than 12 years of age [26]. Thus, in this study, we decided to report the results for the whole age range to increase the statistical power. All traits were adjusted by age and age-squared separately in boys and girls using a linear regression model by Stata statistical package, version 17.0 for Windows (StataCorp, College Station, TX, USA). The linear regression model was also used for statistical testing after correcting the standard errors and confidence intervals (CI) by the cluster option for the lack of statistical independence of twins sampled as pairs [27].

We started the statistical modeling with a factor analysis using the Varimax rotation, which creates uncorrelated (orthogonal) factors, separately in boys and girls. The Eigenvalue statistics suggested a two-factor solution in boys and a three-factor solution in girls. However, in girls, the Eigenvalue for the third factor was only slightly over 1 (1.027), and the factor explained only 5% of the total variance. Thus, we used the two-factor solution in both boys and girls to have comparable results. In this analysis, the first factor explained 67% of the variation in boys and 53% in girls whereas the second factor explained 13 and 20% of the variation in boys and girls, respectively. These factor scores were estimated using the maximum likelihood estimator and then stored as additional variables for the genetic modeling. The factor analysis was conducted using the SPSS statistical software version 28.0 for Windows (IBM Corp, Armonk, NY, USA).

We continued the analyses using genetic twin modeling based on the principle that while MZ twins are virtually genetically identical at the gene sequence level, DZ twins share, on average, half of their segregating genes, as with ordinary siblings [28]. Since the underlying correlation structure between co-twins is known, it is possible to decompose trait variance into genetic and environmental components. Additive genetic variance (A; correlation 1 within MZ and 0.5 within DZ pairs) includes the effects of all loci affecting the trait. Shared environmental variance (C; correlation 1 within both MZ and DZ pairs) includes the effects of all environmental factors making co-twins similar. Unique environmental variation (E; correlation 0 within both MZ and DZ twins) includes the effects of all environmental factors making co-twins dissimilar including measurement error.

We started the genetic modeling with univariate models to find the best fitting model and calculate heritability estimates. Based on co-twin correlations (Supplementary Table 1), we selected the additive genetic/ shared environment/ unique environment (ACE) model as the baseline model. The model fit statistics are presented in Supplementary Table 2. The assumptions of twin modeling were first tested by comparing the fit of the ACE model to the saturated model, which does not make any assumptions but freely estimates all possible statistics. The fit of the ACE model was good; only 6 traits showed poorer fit as compared to the saturated model if using a conventional p-value of 0.05 and none of them was statistically significant if using the Bonferroni corrected p-value for 24 tests (*p* < 0.002). We did not find any evidence for a sex-specific genetic effect, which would be seen as a lower genetic correlation of OSDZ pairs than the 0.5 expected for SSDZ pairs. Additionally, we were able to eliminate the shared environmental component from the model without a statistically significant decrease in the model fit. Thus, we used the additive genetic/ unique environment (AE) model without the sex-specific genetic effect in further analyses; this model showed good fit when compared to the saturated model. However, for some of the traits, we found a decrease in the model fit if using the same estimates for boys and girls. Nevertheless, since this was the case for only a few traits, we presented the genetic modeling results for boys and girls together using the AE model and then compared them to the sex-specific results (see supplementary files).

Using univariate models, we first calculated the proportions of variation explained by additive genetic factors – i.e., (narrow sense) heritability estimates – and unique environmental factors. Then, we utilized bivariate Cholesky decomposition, a model-free method to decompose all variation and covariation in the data into uncorrelated latent factors [29]. This method was used to decompose the covariation between the anthropometric measures into genetic and environmental covariances. Standardizing these covariances provides us the estimates of additive genetic and unique environmental correlations. The genetic twin modeling was conducted using the OpenMx package, version 3.0.2, of R statistical software, estimating the parameters based on the linear structural equations methodology and using the maximum likelihood estimator [30].

## Results

Table 1 presents the descriptive statistics of all anthropometric traits by sex. Girls had thicker skinfolds than boys, whereas boys had broader humerus and femur diameters. Forearm circumference was larger in boys and thigh circumference in girls. Otherwise, the anthropometric measures were roughly similar in boys and girls.

**Table 1:** Means and standard deviations (SD) of anthropometric measures by sex.

	Boys (*N* = 210)		Girls (*N* = 222)		*p*-value of sex difference
	Mean	SD	Mean	SD	
Weight measures					
Weight (kg)	37.6	15.50	37.5	14.95	0.987
BMI (kg/m^2^)	18.4	3.59	18.8	3.76	0.326
Skinfolds (mm)					
Triceps	10.3	4.67	12.9	5.34	<0.0001
Biceps	6.5	3.60	8.5	4.1	<0.0001
Subscapular	8.6	5.6	11.7	7.0	<0.0001
Suprailiac	10.4	7.97	13.4	8.90	0.002
Calf	10.1	5.11	13.5	6.51	<0.0001
Front thigh	15.5	7.05	22.5	9.31	<0.0001
Abdominal	12.8	8.95	17.1	10.64	<0.0001
Circumferences (cm)					
Waist	62.7	9.80	61.9	9.50	0.506
Hip	73.9	12.07	76.4	13.66	0.109
Upper arm	21.0	4.09	21.1	4.12	0.906
Upper arm flexed	22.2	4.26	21.8	4.08	0.367
Forearm	20.6	3.00	19.7	2.93	0.011
Thigh	42.9	8.24	45.5	9.16	0.011
Calf	28.6	4.70	28.7	5.05	0.758
Diameters (cm)					
Biacromial	30.3	4.42	30.0	3.96	0.585
Bicristal	22.0	3.22	22.0	3.28	0.937
Humerus	5.7	0.78	5.3	0.59	<0.0001
Femur	8.2	0.98	7.7	0.81	<0.0001
Height measures (cm)					
Height	139.8	18.99	138.1	17.16	0.427
Sitting height	74.4	8.96	73.5	8.08	0.398

**Fig. 1:**
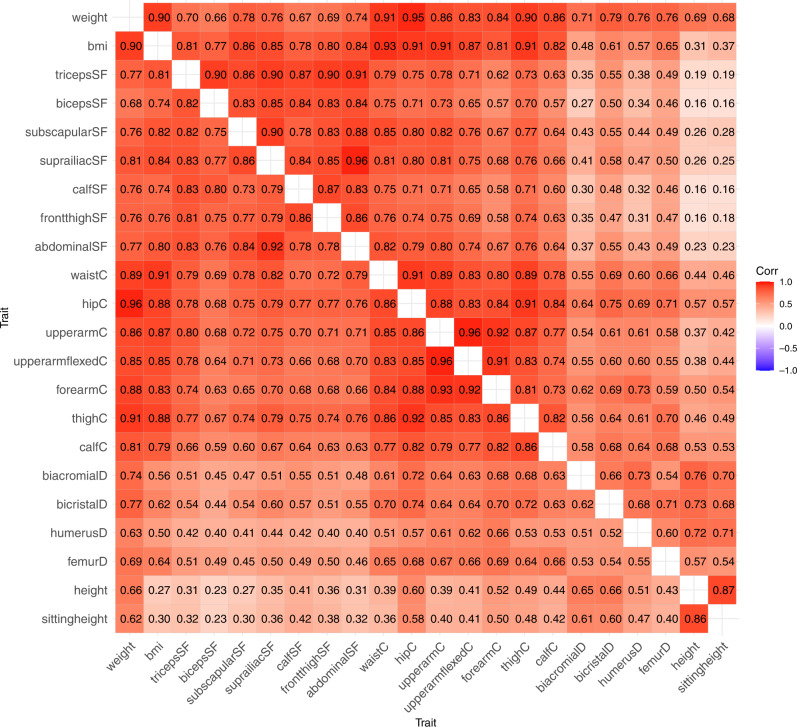
Trait correlations between anthropometric measures in boys (right triangular matrix) and girls (left triangular matrix). SF skinfold, C circumference, D diameter.

Figure 1 presents the correlation matrices between all anthropometric traits in boys (right triangular matrix) and girls (left triangular matrix); the 95% CIs are available in Supplementary Table 3. The correlation structure was roughly similar in boys and girls; only 36 of these 231 correlations showed a *p*-value of sex-difference <0.05 (Supplementary Table 4), which can be because of multiple testing. BMI showed the highest correlations with body circumferences, but the correlations were also high with skinfold thicknesses and somewhat lower with body diameters. On the other hand, height and sitting height showed the highest correlations with body diameters, whereas weaker correlations were found with body circumferences, and they were lowest with skinfold thicknesses.

We then conducted the factor analysis to obtain more insight into the correlation structure of anthropometric measures (Table 2). Obesity measures (BMI, skinfold thicknesses and waist and hip circumferences) and limb circumferences loaded strongly on the first factor, whereas height, sitting height and body diameters loaded strongly on the second factor. However, all anthropometric measures loaded positively on both factors, except height and sitting height showing only weak loadings on the first factor. Communalities were generally high (80% or more for most of the traits) showing that these two factors largely explained the variation of these anthropometric measures. The exceptions were the body diameters in boys and girls and some of the skinfold thicknesses in girls showing only moderate communalities (from 40 to 70%).

**Table 2:** Factor loadings and communalities of anthropometric measures using a two-factor solution in boys and girls^a^.

	First factor		Second factor		Communalities	
	Boys	Girls	Boys	Girls	Boys	Girls
Weight measures						
Weight	0.625	0.818	0.775	0.574	0.991	0.999
BMI	0.830	0.985	0.480	0.164	0.919	0.996
Skinfolds						
Triceps	0.928	0.796	0.153	0.225	0.884	0.684
Biceps	0.887	0.734	0.134	0.142	0.805	0.559
Subscapular	0.876	0.798	0.288	0.185	0.851	0.672
Suprailiac	0.906	0.806	0.25	0.264	0.883	0.719
Calf	0.877	0.696	0.145	0.331	0.791	0.593
Front thigh	0.899	0.726	0.156	0.278	0.832	0.604
Abdominal	0.915	0.778	0.219	0.234	0.885	0.660
Circumferences						
Waist	0.770	0.876	0.547	0.309	0.892	0.863
Hip	0.702	0.809	0.663	0.511	0.932	0.916
Upper arm	0.773	0.856	0.496	0.312	0.843	0.831
Upper arm flexed	0.703	0.832	0.510	0.326	0.754	0.799
Forearm	0.583	0.771	0.625	0.442	0.731	0.790
Thigh	0.719	0.828	0.582	0.414	0.856	0.857
Calf	0.570	0.789	0.647	0.433	0.743	0.811
Diameters						
Biacromial	0.203	0.476	0.760	0.603	0.619	0.589
Bicristal	0.403	0.521	0.709	0.603	0.665	0.636
Humerus	0.249	0.431	0.779	0.469	0.669	0.406
Femur	0.445	0.587	0.673	0.362	0.650	0.476
Height measures						
Height	−0.006	0.097	0.892	0.995	0.796	0.999
Sitting height	0.021	0.158	0.870	0.843	0.756	0.735

^a^Varimax rotation is used.

Next, we conducted the univariate twin modeling for these factor scores and all anthropometric measures in boys and girls (Table 3). Additive genetic variation explained a major part of the variation of all the traits, and the heritability estimates were more than 80% for most of them. The remaining variation was explained by unique environmental factors. In the sex-specific results, we found that the heritability estimates for most of the traits were somewhat higher in boys than in girls (Supplementary Table 5). The largest differences were found for body diameters; however, CIs were also wide in these sex-specific analyses.

**Table 3:** Additive genetic and unique environmental variance components of anthropometric measures and underlying factors in boys and girls.

	Additive genetic factors			Unique environmental factors		
	a^2^	95% confidence intervals		e^2^	95% confidence intervals	
		LL	UL		LL	UL
Weight measures						
Weight	0.89	0.85	0.92	0.11	0.08	0.15
BMI	0.89	0.85	0.92	0.11	0.08	0.15
Skinfolds						
Triceps	0.84	0.78	0.89	0.16	0.11	0.22
Biceps	0.76	0.68	0.83	0.24	0.17	0.32
Subscapular	0.87	0.81	0.91	0.13	0.09	0.19
Suprailiac	0.84	0.77	0.89	0.16	0.11	0.23
Calf	0.79	0.70	0.84	0.21	0.16	0.30
Front thigh	0.85	0.79	0.89	0.15	0.11	0.21
Abdominal	0.83	0.75	0.88	0.17	0.12	0.25
Circumferences						
Waist	0.83	0.76	0.88	0.17	0.12	0.24
Hip	0.89	0.85	0.92	0.11	0.08	0.15
Upper arm	0.74	0.64	0.81	0.26	0.19	0.36
Upper arm flexed	0.67	0.56	0.76	0.33	0.24	0.44
Forearm	0.63	0.51	0.73	0.37	0.27	0.49
Thigh	0.75	0.66	0.82	0.25	0.18	0.34
Calf	0.80	0.72	0.86	0.20	0.14	0.28
Diameters						
Biacromial	0.82	0.75	0.87	0.18	0.13	0.25
Bicristal	0.79	0.71	0.85	0.21	0.15	0.29
Humerus	0.82	0.74	0.87	0.18	0.13	0.26
Femur	0.62	0.49	0.72	0.38	0.28	0.51
Height measures						
Height	0.92	0.88	0.94	0.08	0.06	0.12
Sitting height	0.91	0.88	0.94	0.09	0.06	0.12
Factors						
First factor	0.88	0.82	0.91	0.12	0.09	0.18
Second factor	0.91	0.88	0.94	0.09	0.06	0.12

**Fig. 2:**
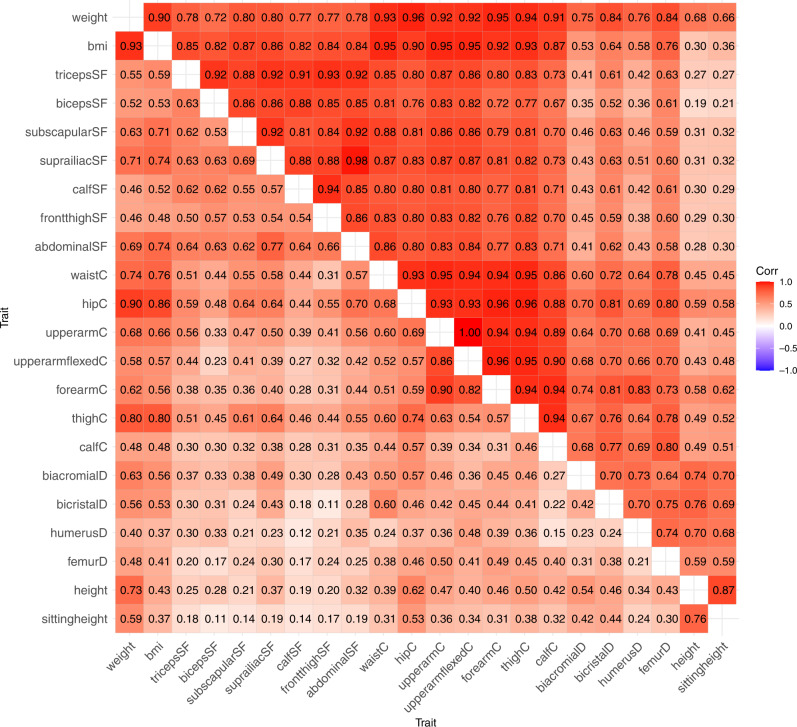
Additive genetic correlations (right triangular matrix) and unique environmental correlations (left triangular matrix) between anthropometric measures in boys and girls. SF skinfold, C circumference, D diameter.

We continued the genetic modeling by analyzing genetic and environmental correlations between the anthropometric traits in boys and girls (Fig. 2; the 95% CIs are available in Supplementary Table 6). Additive genetic correlations (right triangular matrix) were generally high and followed the same pattern as found in the trait correlations. Between the obesity-related traits (BMI, skinfold thicknesses and waist and hip circumferences), the genetic correlations varied between 0.72 and 0.98, indicating that 52 to 96% of the genetic variation is shared between the obesity-related traits. Height and sitting height showed the highest genetic correlations with the body diameter measures, but they were lower than among the obesity measures, i.e., from 0.58 to 0.76, indicating that 34 to 58% of the genetic variation is shared between these traits. The unique environmental correlations (left triangular matrix) were also substantial but remarkably lower than the additive genetic correlations.

Finally, we replicated the analyses in boys and girls to see whether there were any sex differences in these correlation patterns. Supplementary Fig. 1 presents the additive genetic correlations in boys (right triangular matrix) and girls (left triangular matrix); the 95% CIs are available in Supplementary Table 7. We did not find any systematic differences between the genetic correlations in boys and girls. When analyzing the unique environmental correlations, no systematic sex differences were found either (Supplementary Fig. 2; the 95% CIs are available in Supplementary Table 8).

## Discussion

In this comprehensive twin study of 22 anthropometric traits, we found that the same genetic factors underlined the different anthropometric traits traditionally used to measure obesity, namely, BMI, skinfold thicknesses and waist and hip circumferences. Based on the genetic correlations, we estimated that from half to nearly all the genetic variation was shared between these different obesity-related traits. Further, the heritability estimates for these traits were high: genetic factors explained from 80 to 90% of the variation for most of them. Thus, there is a set of genes explaining a substantial proportion of variation of different obesity-related traits in children. A large number of loci associated with childhood BMI have been identified in a GWA study and are also associated with obesity measures in adulthood [18]. Thus, these loci may also underlie the variation of other obesity-related traits. We also identified genetic correlations of height with body diameters and somewhat lower correlations with body circumferences and skinfold thicknesses. Thousands of genetic variants have been identified for height in a GWA study [8]. Thus, it would be important to study how these genetic variants are associated with other anthropometric traits.

The underlying mechanisms behind the genetic correlations are poorly known and can vary between the traits. The genetic variants for height and BMI have largely similar associations within sibling pairs as those found at the population level, suggesting that they affect independently of family environment [31]. The genetic variants associated with higher BMI have been found to be enriched in the brain, especially in the hypothalamus, pituitary gland, hippocampus and limbic system [9, 32, 33]. These brain areas are important in appetite regulation, learning, cognition, emotion and memory [34]. Together with the previous direct evidence on the genetic component behind eating behavior [22], these results suggest that the genetic factors underlying covariation between different obesity traits can partly be associated with energy intake. However, it is also noteworthy that even when high, the genetic correlations between most obesity-related traits were much less than 1, suggesting that different genetic factors also affect different obesity measures. There is evidence from a GWA study that the genetic variants associated with body fat distribution are related to lipid metabolism and adipose tissue regulation in particular [35]. On the other hand, the expression of genes associated with height have been found to be enriched in growth plate chondrocytes [36]. It is interesting to note that there are genetic correlations between height and anthropometric traits not related to the ossification of bones, such as skinfold thicknesses, which is also consistent with a previous family study [17]. Thus, it is likely that part of the genes associated with height affect through other mechanisms and may, for example, reflect nutrition choices that promote both weight gain and height growth. These associations can have a basis starting from fetal life when the same genes regulate the development of different body parts [20], but these molecular level mechanisms are complex and still poorly understood [37]. More studies are thus needed to identify these different pathways from genes to various anthropometric traits.

The correlation pattern between different anthropometric traits suggests that it is possible to create summary scales capturing body morphology variation. The best known of these scales is probably the somatotype, based on 10 anthropometric measures and classifying the physique or body form through three specific components that characterize the configuration of the body: endomorphy (relative fatness), mesomorphy (relative musculoskeletal development) and ectomorphy (relative linearity) [38]. In our previous study based on these same data, we found that these somatotype components showed high heritability [26]. In this current study, we found that a large part of the variation and covariation of 22 anthropometric traits can be captured by two orthogonal factors. In particular, obesity-related traits (BMI, skinfold thicknesses and waist and hip circumferences) were loaded on the first factor. However, the loadings on this factor were also high for limb circumferences, which are a combination of bone, muscle, and fat tissues. Height, sitting height and body diameters loaded strongly on the second factor, but substantial loadings were also found for all body circumferences. Thus, we could interpret that the first factor reflects body fatness and the second factor body tallness/robustness. Both factors showed high heritability. It is well known that excess body fat is associated with higher [39], and body tallness with lower [40], risk of cardiovascular diseases, and therefore a better understanding of the biological background of these factors may have important public health implications. On the other hand, the high correlations between different obesity measures suggest that they largely capture the same information. Thus, the detailed anthropometric measures increase the accuracy when measuring body fatness and underlying genetic susceptibility. However, if detailed measures are not possible, such as in large epidemiological studies, only one measure may be enough to offer sufficient information on obesity.

We found that the heritability estimates were higher for most of the anthropometric traits in boys than in girls. This sexual dimorphism parallels the findings of a large pooled twin study in that heritability estimates of BMI were systematically higher in boys than in girls over childhood [6]; for height, the results were somewhat less systematic but also showed higher heritability in boys at most of the ages [7]. These results may suggest that the female body shows more environmental plasticity as compared to the male body. The sexual dimorphism of phenotype environmental plasticity is very common in the animal kingdom, but it is affected by traits such as evolutionary pressure and cross-sex genetic correlations [41]. Thus, more studies are needed to analyze this issue in humans to discover whether this reflects, for example, evolutionary pressure for the female body to better adapt to the changing environment. However, in light of these results, it is interesting to note that all correlations (i.e., trait correlations, additive genetic correlations and unique environmental correlations) between these anthropometric traits were similar in boys and girls. This suggests that despite the somewhat different role of genetic and environmental factors behind the variation of anthropometric traits, the pleotropic effects behind body size and morphology are roughly similar in both sexes.

Our study has both strengths and limitations. Our main strength is the very detailed measures of the human body – 22 anthropometric traits together – in a twin data set allowing us to analyze the genetic regulation of human body morphology in detail. In addition, genetic studies in Southern European populations are rare compared to Northern European and North American populations of European ancestry. Our main limitation is that the sample size was not large enough to study potential differences over the age range studied. For example, in a very large twin study pooling data from several cohorts, environmental factors shared by co-twins affected BMI variation in early childhood but its effect disappeared in adolescence [6]. In our previous study, we analyzed the heritability estimates of 10 of the traits also used in the current study and found no systematic differences between children younger or older than 12 years of age [26]. However, separating shared environmental effects from additive genetic effects requires considerable statistical power [42]. Thus, shared environmental factors may also affect anthropometric traits in early childhood in our data, but because of lack of power, we cannot identify these factors and their effect is thus pooled with additive genetic factors. Furthermore, the cross-sectional data do not allow analyzing developmental trajectories and, for example, studying whether the same genetic factors affect these anthropometric traits at different ages. To study these issues, larger studies, preferably with follow up data over childhood, are needed. Finally, we had only anthropometric measures and not dual-energy X-ray absorptiometry (DEXA), bioimpedance, computer tomography or other measures of body composition allowing us to directly assess fat and fat free mass. This information would have allowed us to calculate genetic correlations between fat mass, fat free mass and different anthropometric measures. However, considering the high genetic correlations between obesity related anthropometric traits in our data, we can speculate that the genetic correlations between fat mass and these anthropometric traits would also be high.

In conclusion, the correlation structure of detailed anthropometric measures suggested that there are two factors – general body fatness and body height/robustness of the skeleton – underlying body morphology. In particular, body fatness measures showed high genetic correlations suggesting that there is a set of genes affecting overall body fatness. These genetic variants common for various anthropometric traits probably play an important role in the formation of human body size and morphology. Considering the role of obesity and other human physique features behind metabolic and many other chronic diseases, a better understanding on these pleiotropic effects can also shed more light on individual variation in health risk profiles.

## Supplementary information

Supplemental material

## Data Availability

The data that support the findings of this study are available from the Madeira Family Study, but restrictions apply to the availability of these data, which were used under license for the current study, and thus are not publicly available. Data are, however, available from the authors upon reasonable request and with permission of Duarte Freitas (dfreitas@staff.uma.pt), the PI of the Madeira Family Study.

## Code availability

All computer codes are available from the corresponding author.
