# Supplementary material for: Genetic regulation of body size and morphology in children: a twin study of 22 anthropometric traits
Source: Int J Obes (Lond). 2023 Jan 12;47(3):181–9. doi: 10.1038/s41366-023-01253-0 (PMC10023566; doi:10.1038/s41366-023-01253-0)
Supplement: Supplementary file 1 — Supplemental material [file 41366_2023_1253_MOESM1_ESM.pdf]

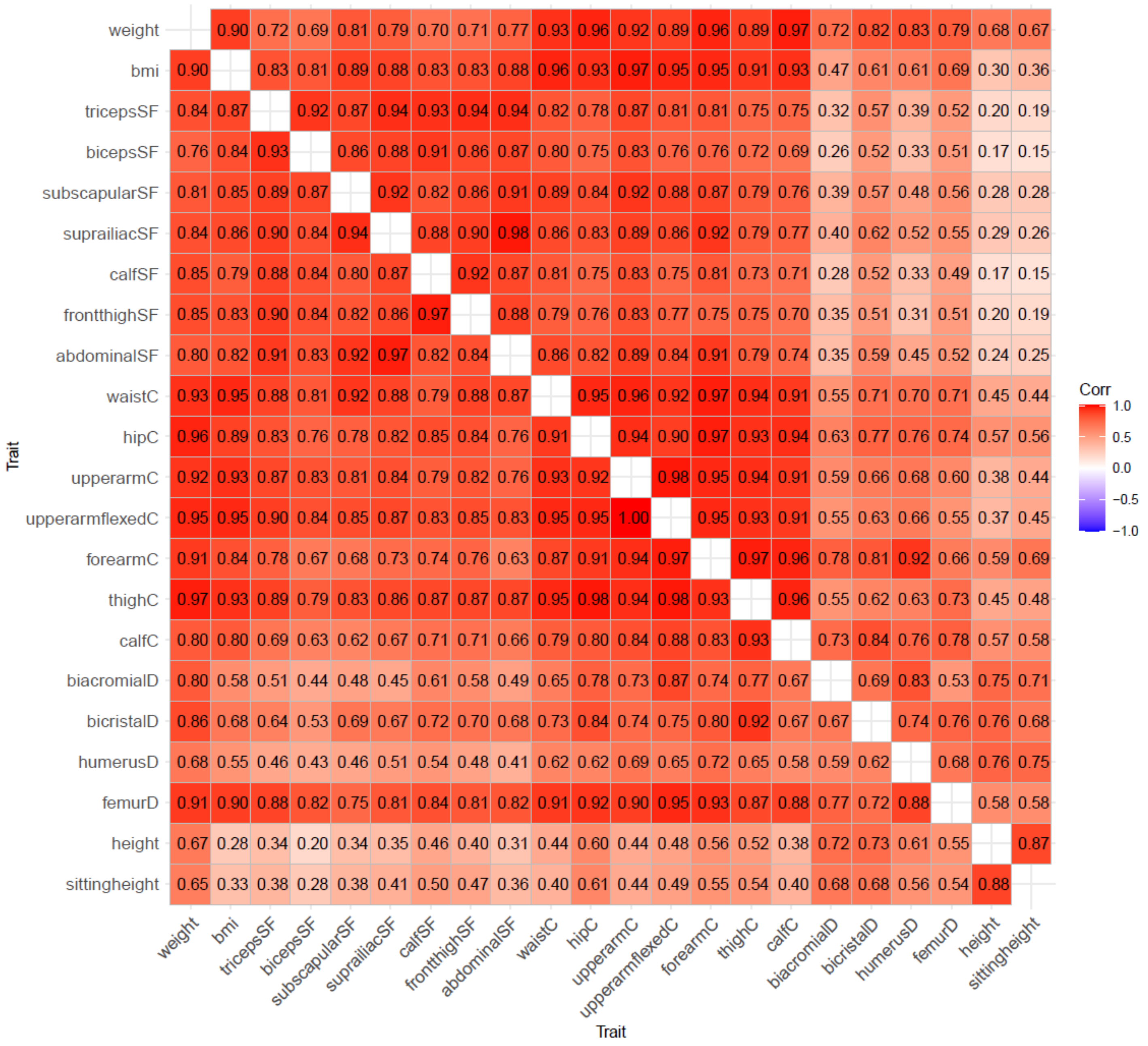

Supplementary figure 1. Additive genetic correlations between anthropometric measures in boys (right triangular matrix) and girls (left triangular matrix). Abbreviations: SF=skinfold, C=circumference, D=diameter

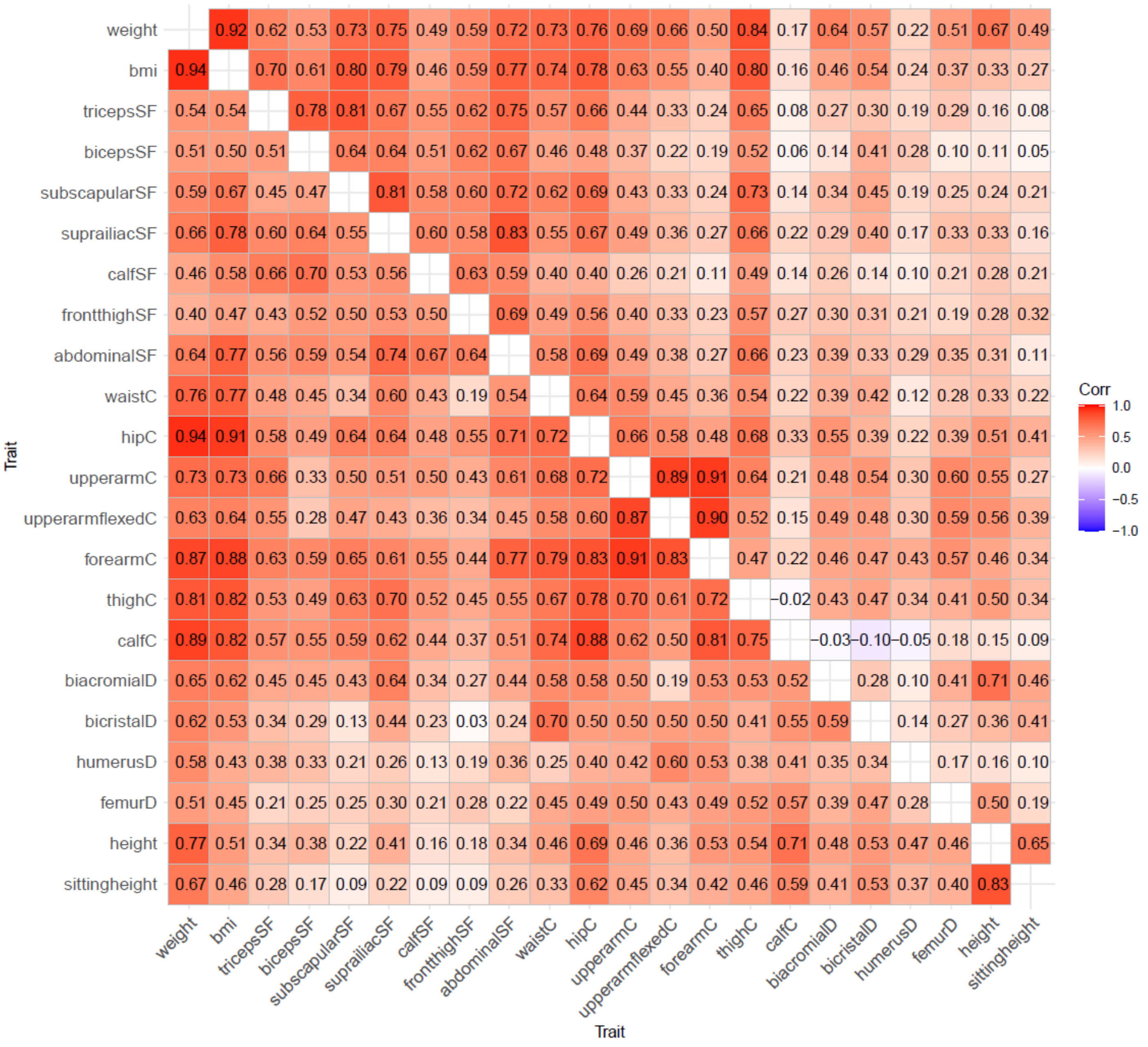

Supplementary figure 2. Unique environmental correlations between anthropometric measures in boys (right triangular matrix) and girls (left triangular matrix). Abbreviations: SF=skinfold, C=circumference, D=diameter

Supplementary table 1. Twin correlations of anthropometric measures and underlying factors by sex and zygosity.

	Monozygotic		Same-sex dizygotic		Opposite-sex dizygotic
	**Boys**	**Girls**	**Boys**	**Girls**	
**Weight measures**					
Weight	0.95	0.82	0.53	0.21	0.28
BMI	0.94	0.84	0.54	0.26	0.29
**Skinfolds**					
Triceps	0.86	0.82	0.43	0.36	0.44
Biceps	0.85	0.67	0.35	0.44	0.43
Subscapular	0.85	0.89	0.35	0.17	0.33
Suprailiac	0.86	0.80	0.29	0.19	0.42
Calf	0.83	0.75	0.39	0.44	0.44
Front thigh	0.89	0.79	0.52	0.29	0.48
Abdominal	0.86	0.75	0.41	0.32	0.37
**Circumferences**					
Waist	0.89	0.75	0.47	0.39	0.20
Hip	0.94	0.83	0.55	0.27	0.33
Upper arm	0.75	0.74	0.52	0.39	0.32
Upper arm flexed	0.73	0.62	0.48	0.32	0.37
Forearm	0.55	0.79	0.52	0.25	0.32
Thigh	0.92	0.63	0.53	0.32	0.28
Calf	0.81	0.81	0.68	0.17	0.27
**Diameters**					
Biacromial	0.89	0.71	0.51	0.50	0.19
Bicristal	0.93	0.69	0.26	0.31	0.26
Humerus	0.89	0.73	0.56	0.30	0.26
Femur	0.91	0.28	0.32	0.37	0.10
**Height measures**					
Height	0.96	0.84	0.48	0.44	0.47
Sitting height	0.95	0.84	0.58	0.29	0.44
**Factors**					
First factor	0.89	0.85	0.33	0.28	0.40
Second factor	0.96	0.85	0.58	0.49	0.29

Supplementary table 2. Model fit statistics of anthropometric measures and underlying factors comparing different genetic models.

	**Saturated model**		**Full ACE model[1]**		**ACE model without sex-specific genetic effect[2]**		**AE model without sex-specific genetic effect[3]**		**AE model without sex-specific genetic effect[4]**		**AE model with same parameter estimates for boys and girls[5]**	
	**-2 LL**	**d.f.**	**Δ -2 LL**	**p-value**	**Δ -2 LL**	**p-value**	**Δ -2 LL**	**p-value**	**Δ -2 LL**	**p-value**	**Δ -2 LL**	**p-value**
**Weight measures**												
Weight	1648.84	407	28.35	0.0287	0.61	0.4338	1.50	0.4712	30.47	0.0461	8.74	0.0126
BMI	1422.88	407	20.52	0.1979	0.45	0.5044	1.85	0.3971	22.81	0.2459	6.57	0.0374
**Skinfolds**												
Triceps	2235.35	407	14.70	0.5466	0.33	0.5636	0.00	1.0000	15.03	0.7204	1.97	0.3730
Biceps	2343.38	407	12.56	0.7048	0.26	0.6080	0.04	0.9796	12.86	0.8456	4.71	0.0949
Subscapular	2378.34	407	26.98	0.0417	0.83	0.3620	0.10	0.9520	27.91	0.0851	0.45	0.7983
Suprailiac	2618.48	407	15.03	0.5226	0.02	0.8895	0.00	1.0000	15.05	0.7196	0.81	0.6656
Calf	2339.52	407	13.95	0.6028	0.28	0.5955	0.08	0.9619	14.30	0.7657	1.88	0.3903
Front thigh	2236.06	407	16.64	0.4090	0.21	0.6468	0.09	0.9560	16.94	0.5937	3.33	0.1896
Abdominal	2605.13	407	13.88	0.6076	0.18	0.6743	0.02	0.9915	14.07	0.7793	2.15	0.3408
**Circumferences**												
Waist	1151.04	407	29.38	0.0215	1.04	0.3088	2.57	0.2769	0.2769	0.0241	2.45	0.2939
Hip	2983.40	407	28.59	0.0268	0.29	0.5889	1.11	0.5734	0.5734	0.0518	5.22	0.0735
Upper arm	2046.76	407	22.50	0.1277	0.00	0.9693	1.32	0.5176	0.5176	0.2031	0.98	0.6117
Upper arm flex	2047.24	407	19.79	0.2298	0.02	0.8793	0.48	0.7885	0.7885	0.3774	4.28	0.1176
Forearm	1601.05	403	26.29	0.0501	0.52	0.4717	2.24	0.3258	0.3258	0.0652	12.79	0.0017
Thigh	2545.54	403	18.87	0.2754	0.00	0.9486	0.33	0.8500	0.8500	0.4441	24.49	0.0000
Calf	2038.43	405	25.45	0.0623	0.05	0.8205	7.07	0.0292	0.0292	0.0269	1.39	0.4983
**Diameters**												
Biacromial	1619.61	407	15.09	0.5183	3.61	0.0573	0.08	0.9631	18.78	0.4713	5.52	0.0632
Bicristal	1493.40	407	27.38	0.0374	0.01	0.9389	0.00	1.0000	27.39	0.0960	14.25	0.0008
Humerus	2120.27	403	22.79	0.1194	0.01	0.9144	2.74	0.2543	25.54	0.1435	6.44	0.0400
Femur	2615.16	405	28.12	0.0306	0.05	0.8237	2.40	0.3015	30.56	0.0450	35.53	0.0000
**Height measures**												
Height	2634.15	407	16.68	0.4064	0.44	0.5078	0.00	1.0000	17.12	0.5816	8.34	0.0155

Sitting height	2108.54	407	12.58	0.7029	0.27	0.6047	0.58	0.7479	13.43	0.8157	9.89	0.0071
**Factors**												
First factor	1056.46	402	12.17	0.7321	0.10	0.7483	0.00	1.0000	12.28	0.8735	1.17	0.5557
Second factor	990.77	402	21.49	0.1604	0.05	0.8162	0.69	0.7089	22.23	0.2728	12.41	0.0020

Abbreviations: -2LL (-2 log-likelihood); d.f. (degrees of freedom); Δ (change); ACE (additive genetic/ shared environment/ unique environment) model; AE (additive genetic/ unique environment) model

[1]Compared to the saturated model (Δ d.f. 16); [2]Compared to the full ACE model (Δ d.f. 1); [3]Compared to the ACE model without sex-specific genetic effect (Δ d.f. 1); [4]Compared to the saturated model (Δ d.f. 19); [5]Compared to the AE model without sex-specific genetic effect (Δ d.f. 2)

Supplementary table 3. Trait correlations with 95% confidence intervals of anthropometric measures in boys (right triangular matrix) and girls (left triangular matrix).

	1	2	3	4	5	6	7	8	9	10	11	12	13	14	15	16	17	18	19	20	21	22
**1**		0.90 0.87, 0.92	0.70 0.62, 0.76	0.66 0.58, 0.73	0.78 0.72, 0.82	0.76 0.70, 0.82	0.67 0.58, 0.74	0.69 0.61, 0.75	0.74 0.68, 0.80	0.91 0.88, 0.93	0.95 0.94, 0.96	0.86 0.82, 0.89	0.83 0.78, 0.87	0.84 0.79, 0.88	0.90 0.87, 0.92	0.86 0.82, 0.89	0.71 0.64, 0.77	0.79 0.74, 0.84	0.76 0.70, 0.81	0.76 0.70, 0.81	0.69 0.61, 0.75	0.68 0.60, 0.75
**2**	0.90 0.87, 0.92		0.81 0.76, 0.85	0.77 0.71, 0.82	0.86 0.83, 0.89	0.85 0.80, 0.88	0.78 0.72, 0.83	0.80 0.75, 0.85	0.84 0.80, 0.88	0.93 0.91, 0.95	0.91 0.88, 0.93	0.91 0.89, 0.93	0.87 0.83, 0.90	0.81 0.76, 0.85	0.91 0.89, 0.93	0.82 0.77, 0.86	0.48 0.37, 0.58	0.61 0.51, 0.69	0.57 0.47, 0.66	0.65 0.57, 0.72	0.31 0.18, 0.42	0.37 0.25, 0.48
**3**	0.77 0.72, 0.82	0.81 0.76, 0.85		0.90 0.87, 0.92	0.86 0.82, 0.89	0.90 0.87, 0.92	0.87 0.83, 0.90	0.90 0.87, 0.92	0.91 0.88, 0.93	0.79 0.73, 0.83	0.75 0.69, 0.81	0.78 0.72, 0.83	0.71 0.64, 0.77	0.62 0.53, 0.70	0.73 0.66, 0.79	0.63 0.54, 0.70	0.35 0.22, 0.46	0.55 0.44, 0.63	0.38 0.26, 0.49	0.49 0.38, 0.59	0.19 0.05, 0.31	0.19 0.05, 0.31
**4**	0.68 0.61, 0.75	0.74 0.68, 0.80	0.82 0.77, 0.86		0.83 0.79, 0.87	0.85 0.80, 0.88	0.84 0.80, 0.88	0.83 0.78, 0.87	0.84 0.80, 0.88	0.75 0.69, 0.81	0.71 0.64, 0.77	0.73 0.66, 0.79	0.65 0.57, 0.72	0.57 0.47, 0.66	0.70 0.62, 0.76	0.57 0.47, 0.65	0.27 0.14, 0.39	0.50 0.39, 0.59	0.34 0.22, 0.46	0.46 0.34, 0.56	0.16 0.03, 0.29	0.16 0.03, 0.29
**5**	0.76 0.70, 0.81	0.82 0.77, 0.86	0.82 0.77, 0.86	0.75 0.69, 0.80		0.90 0.87, 0.92	0.78 0.73, 0.83	0.83 0.78, 0.86	0.88 0.85, 0.91	0.85 0.81, 0.88	0.80 0.75, 0.85	0.82 0.77, 0.86	0.76 0.70, 0.81	0.67 0.59, 0.74	0.77 0.71, 0.82	0.64 0.55, 0.71	0.43 0.31, 0.53	0.55 0.45, 0.64	0.44 0.32, 0.54	0.49 0.38, 0.59	0.26 0.13, 0.38	0.28 0.15, 0.40
**6**	0.81 0.76, 0.85	0.84 0.79, 0.87	0.83 0.79, 0.87	0.77 0.71, 0.82	0.86 0.82, 0.89		0.84 0.79, 0.87	0.85 0.81, 0.88	0.96 0.94, 0.97	0.81 0.76, 0.85	0.80 0.74, 0.84	0.81 0.75, 0.85	0.75 0.69, 0.81	0.68 0.60, 0.75	0.76 0.70, 0.81	0.66 0.57, 0.73	0.41 0.29, 0.52	0.58 0.49, 0.67	0.47 0.35, 0.57	0.50 0.39, 0.59	0.26 0.13, 0.39	0.25 0.12, 0.37
**7**	0.76 0.70, 0.81	0.74 0.67, 0.79	0.83 0.78, 0.87	0.80 0.74, 0.84	0.73 0.67, 0.79	0.79 0.74, 0.84		0.87 0.83, 0.90	0.83 0.78, 0.86	0.75 0.68, 0.80	0.71 0.63, 0.77	0.71 0.64, 0.77	0.65 0.56, 0.72	0.58 0.48, 0.66	0.71 0.63, 0.77	0.60 0.51, 0.68	0.30 0.18, 0.42	0.48 0.37, 0.58	0.32 0.19, 0.43	0.46 0.34, 0.56	0.16 0.02, 0.29	0.16 0.03, 0.29
**8**	0.76 0.69, 0.81	0.76 0.70, 0.81	0.81 0.76, 0.85	0.75 0.69, 0.80	0.77 0.70, 0.81	0.79 0.73, 0.83	0.86 0.82, 0.89		0.86 0.82, 0.89	0.76 0.69, 0.81	0.74 0.67, 0.79	0.75 0.68, 0.80	0.69 0.61, 0.75	0.58 0.49, 0.67	0.74 0.67, 0.80	0.63 0.54, 0.70	0.35 0.23, 0.47	0.47 0.36, 0.57	0.31 0.18, 0.43	0.47 0.36, 0.57	0.16 0.03, 0.29	0.18 0.04, 0.31
**9**	0.77 0.71, 0.82	0.80 0.75, 0.84	0.83 0.78, 0.86	0.76 0.69, 0.81	0.84 0.79, 0.87	0.92 0.90, 0.94	0.78 0.72, 0.83	0.78 0.72, 0.82		0.82 0.77, 0.86	0.79 0.73, 0.84	0.80 0.74, 0.84	0.74 0.68, 0.80	0.67 0.58, 0.74	0.76 0.69, 0.81	0.64 0.55, 0.71	0.37 0.25, 0.49	0.55 0.44, 0.64	0.43 0.32, 0.54	0.49 0.38, 0.58	0.23 0.10, 0.35	0.23 0.10, 0.36
**10**	0.89 0.86, 0.91	0.91 0.89, 0.93	0.79 0.74, 0.84	0.69 0.61, 0.75	0.78 0.73, 0.83	0.82 0.77, 0.86	0.70 0.62, 0.76	0.72 0.65, 0.78	0.79 0.74, 0.84		0.91 0.88, 0.93	0.89 0.85, 0.91	0.83 0.78, 0.87	0.80 0.75, 0.85	0.89 0.86, 0.91	0.78 0.73, 0.83	0.55 0.44, 0.63	0.69 0.61, 0.75	0.60 0.50, 0.68	0.66 0.57, 0.73	0.44 0.32, 0.54	0.46 0.34, 0.56
**11**	0.96 0.94, 0.98	0.88 0.84, 0.90	0.78 0.72, 0.82	0.68 0.61, 0.75	0.75 0.68, 0.80	0.79 0.74, 0.84	0.77 0.71, 0.82	0.77 0.71, 0.82	0.76 0.70, 0.81	0.86 0.82, 0.89		0.88 0.85, 0.91	0.83 0.78, 0.87	0.84 0.80, 0.88	0.91 0.89, 0.93	0.84 0.80, 0.88	0.64 0.55, 0.71	0.75 0.68, 0.80	0.69 0.61, 0.76	0.71 0.63, 0.77	0.57 0.47, 0.66	0.57 0.47, 0.65
**12**	0.86 0.82, 0.89	0.87 0.83, 0.90	0.80 0.75, 0.85	0.68 0.60, 0.74	0.72 0.64, 0.77	0.75 0.69, 0.80	0.70 0.62, 0.76	0.71 0.64, 0.77	0.71 0.64, 0.77	0.85 0.81, 0.89	0.86 0.83, 0.89		0.96 0.94, 0.97	0.92 0.89, 0.94	0.87 0.83, 0.90	0.77 0.70, 0.82	0.54 0.43, 0.63	0.61 0.52, 0.69	0.61 0.52, 0.69	0.58 0.48, 0.66	0.37 0.25, 0.48	0.42 0.30, 0.52
**13**	0.85 0.80, 0.88	0.85 0.81, 0.88	0.78 0.73, 0.83	0.64 0.55, 0.71	0.71 0.64, 0.77	0.73 0.66, 0.78	0.66 0.58, 0.73	0.68 0.61, 0.75	0.70 0.62, 0.76	0.83 0.79, 0.87	0.85 0.80, 0.88	0.96 0.94, 0.97		0.91 0.88, 0.93	0.83 0.78, 0.86	0.74 0.67, 0.80	0.55 0.45, 0.64	0.60 0.51, 0.68	0.60 0.51, 0.68	0.55 0.45, 0.64	0.38 0.25, 0.49	0.44 0.32, 0.54

**14**	0.88 0.85, 0.91	0.83 0.79, 0.87	0.74 0.67, 0.79	0.63 0.54, 0.70	0.65 0.57, 0.72	0.70 0.62, 0.76	0.68 0.60, 0.74	0.68 0.60, 0.74	0.66 0.58, 0.73	0.84 0.80, 0.88	0.88 0.85, 0.91	0.93 0.91, 0.94	0.92 0.89, 0.94		0.81 0.75, 0.85	0.73 0.66, 0.79	0.62 0.53, 0.70	0.69 0.61, 0.75	0.73 0.66, 0.79	0.59 0.49, 0.67	0.50 0.39, 0.59	0.54 0.44, 0.63
**15**	0.91 0.89, 0.93	0.88 0.85, 0.91	0.77 0.71, 0.82	0.67 0.59, 0.74	0.74 0.67, 0.79	0.79 0.74, 0.84	0.75 0.68, 0.80	0.74 0.67, 0.79	0.76 0.69, 0.81	0.86 0.82, 0.89	0.92 0.90, 0.94	0.85 0.81, 0.89	0.83 0.79, 0.87	0.86 0.82, 0.89		0.82 0.77, 0.86	0.56 0.46, 0.65	0.64 0.55, 0.71	0.61 0.52, 0.69	0.70 0.63, 0.76	0.46 0.35, 0.56	0.49 0.38, 0.59
**16**	0.81 0.76, 0.85	0.79 0.73, 0.83	0.66 0.58, 0.73	0.59 0.50, 0.67	0.60 0.51, 0.68	0.67 0.59, 0.74	0.64 0.55, 0.71	0.63 0.54, 0.70	0.63 0.54, 0.70	0.77 0.71, 0.82	0.82 0.77, 0.86	0.79 0.73, 0.83	0.77 0.70, 0.82	0.82 0.76, 0.86	0.86 0.82, 0.89		0.58 0.48, 0.66	0.68 0.60, 0.75	0.64 0.55, 0.71	0.68 0.60, 0.75	0.53 0.42, 0.62	0.53 0.42, 0.62
**17**	0.74 0.67, 0.79	0.56 0.47, 0.65	0.51 0.40, 0.60	0.45 0.34, 0.55	0.47 0.36, 0.56	0.51 0.40, 0.60	0.55 0.45, 0.64	0.51 0.40, 0.60	0.48 0.37, 0.58	0.61 0.52, 0.68	0.72 0.65, 0.78	0.64 0.56, 0.71	0.63 0.54, 0.70	0.68 0.60, 0.74	0.68 0.60, 0.74	0.63 0.55, 0.71		0.66 0.57, 0.73	0.73 0.66, 0.79	0.54 0.43, 0.63	0.76 0.69, 0.81	0.70 0.62, 0.76
**18**	0.77 0.71, 0.82	0.62 0.53, 0.70	0.54 0.44, 0.63	0.44 0.32, 0.54	0.54 0.44, 0.63	0.60 0.51, 0.68	0.57 0.47, 0.65	0.51 0.40, 0.60	0.55 0.45, 0.63	0.70 0.63, 0.76	0.74 0.68, 0.80	0.64 0.55, 0.71	0.64 0.56, 0.71	0.70 0.63, 0.76	0.72 0.65, 0.78	0.63 0.54, 0.70	0.62 0.53, 0.70		0.68 0.60, 0.74	0.71 0.63, 0.77	0.73 0.66, 0.79	0.68 0.60, 0.74
**19**	0.63 0.54, 0.70	0.50 0.40, 0.60	0.42 0.31, 0.52	0.40 0.28, 0.50	0.41 0.29, 0.51	0.44 0.33, 0.54	0.42 0.30, 0.52	0.40 0.28, 0.50	0.40 0.28, 0.51	0.51 0.40, 0.60	0.57 0.47, 0.65	0.61 0.52, 0.69	0.62 0.53, 0.70	0.66 0.58, 0.73	0.53 0.43, 0.62	0.53 0.42, 0.62	0.51 0.41, 0.60	0.52 0.42, 0.61		0.60 0.50, 0.68	0.72 0.65, 0.78	0.71 0.64, 0.77
**20**	0.69 0.61, 0.75	0.64 0.55, 0.71	0.51 0.40, 0.60	0.49 0.38, 0.58	0.45 0.34, 0.55	0.50 0.39, 0.59	0.49 0.38, 0.58	0.50 0.39, 0.59	0.46 0.35, 0.56	0.65 0.57, 0.72	0.68 0.60, 0.75	0.67 0.59, 0.74	0.66 0.58, 0.73	0.69 0.62, 0.76	0.64 0.56, 0.71	0.66 0.58, 0.73	0.53 0.43, 0.62	0.54 0.44, 0.63	0.55 0.45, 0.63		0.57 0.47, 0.66	0.54 0.43, 0.63
**21**	0.66 0.58, 0.73	0.27 0.14, 0.39	0.31 0.18, 0.42	0.23 0.10, 0.35	0.27 0.15, 0.39	0.35 0.23, 0.46	0.41 0.30, 0.52	0.36 0.24, 0.47	0.31 0.19, 0.43	0.39 0.28, 0.50	0.60 0.50, 0.67	0.39 0.28, 0.50	0.41 0.29, 0.51	0.52 0.42, 0.61	0.49 0.39, 0.59	0.44 0.32, 0.54	0.65 0.57, 0.72	0.66 0.57, 0.72	0.51 0.41, 0.60	0.43 0.31, 0.53		0.87 0.84, 0.90
**22**	0.62 0.53, 0.70	0.30 0.18, 0.42	0.32 0.20, 0.44	0.23 0.10, 0.35	0.30 0.17, 0.41	0.36 0.24, 0.47	0.42 0.30, 0.52	0.38 0.26, 0.49	0.32 0.19, 0.43	0.36 0.24, 0.47	0.58 0.48, 0.66	0.40 0.28, 0.50	0.41 0.29, 0.51	0.50 0.40, 0.59	0.48 0.38, 0.58	0.42 0.30, 0.52	0.61 0.53, 0.69	0.60 0.51, 0.68	0.47 0.36, 0.56	0.40 0.29, 0.51	0.86 0.82, 0.89	

Note: 1=weight, 2=bmi, 3=tricepsSF, 4=bicepsSF, 5=subscapularSF, 6=suprailiacSF, 7=calfSF, 8=frontthighSF, 9=abdominalSF, 10=waistC, 11=hipC, 12=upperarmC, 13=upper armflexedC, 14=forearmC, 15=thighC, 16=calfC, 17=biacromialD, 18=bicristalD, 19=humerusD, 20=femurD, 21=height, 22=sitting height (SF=skinfold; C=circumference; D=diameter)

Supplementary table 4. Statistical significance (p-values) of sex difference of the correlations between anthropometric traits.

	1	2	3	4	5	6	7	8	9	10	11	12	13	14	15	16	17	18	19	20	21
**1**																					
**2**	0.919																				
**3**	0.091	0.998																			
**4**	0.711	0.416	0.001																		
**5**	0.700	0.086	0.103	0.022																	
**6**	0.208	0.768	0.008	0.021	0.129																
**7**	0.044	0.319	0.111	0.151	0.212	0.185															
**8**	0.141	0.230	<0.0001	0.030	0.091	0.050	0.673														
**9**	0.584	0.210	<0.0001	0.013	0.068	0.002	0.193	0.012													
**10**	0.371	0.198	0.911	0.163	0.039	0.813	0.303	0.472	0.468												
**11**	0.718	0.146	0.532	0.572	0.139	0.902	0.183	0.407	0.439	0.027											
**12**	0.888	0.034	0.514	0.243	0.008	0.166	0.714	0.344	0.037	0.148	0.412										
**13**	0.538	0.469	0.099	0.811	0.243	0.568	0.769	0.925	0.348	0.890	0.561	0.866									
**14**	0.071	0.490	0.030	0.333	0.769	0.772	0.108	0.110	0.905	0.230	0.076	0.460	0.460								
**15**	0.416	0.103	0.399	0.613	0.405	0.449	0.415	0.967	0.944	0.229	0.538	0.541	0.762	0.088							
**16**	0.109	0.409	0.539	0.717	0.484	0.847	0.533	0.947	0.860	0.684	0.393	0.544	0.572	0.035	0.165						
**17**	0.583	0.252	0.044	0.035	0.619	0.192	0.002	0.051	0.173	0.351	0.132	0.101	0.193	0.331	0.048	0.359					
**18**	0.578	0.818	0.962	0.429	0.913	0.778	0.188	0.631	0.980	0.714	0.917	0.648	0.493	0.789	0.122	0.335	0.549				
**19**	0.007	0.320	0.626	0.521	0.741	0.778	0.221	0.319	0.696	0.178	0.031	0.956	0.790	0.179	0.225	0.086	<0.0001	0.012			
**20**	0.127	0.787	0.810	0.696	0.535	0.957	0.665	0.735	0.773	0.940	0.602	0.138	0.080	0.066	0.260	0.712	0.981	0.006	0.416		
**21**	0.573	0.686	0.173	0.460	0.849	0.323	0.004	0.027	0.346	0.593	0.713	0.788	0.713	0.742	0.678	0.211	0.031	0.119	<0.0001	0.042	
**22**	0.286	0.416	0.128	0.445	0.839	0.207	0.003	0.021	0.349	0.224	0.918	0.807	0.675	0.567	0.888	0.138	0.128	0.190	<0.0001	0.077	0.483

Note: 1=weight, 2=bmi, 3=tricepsSF, 4=bicepsSF, 5=subscapularSF, 6=suprailiacSF, 7=calfSF, 8=frontthighSF, 9=abdominalSF, 10=waistC, 11=hipC, 12=upperarmC, 13=upper armflexedC, 14=forearmC, 15=thighC, 16=calfC, 17=biacromialD, 18=bicristalD, 19=humerusD, 20=femurD, 21=height, 22=sitting height (SF=skinfold; C=circumference; D=diameter)

Supplementary table 5. Additive genetic and unique environmental variance components with 95% confidence intervals of anthropometric measures and underlying factors stratified by sex.

	Boys						Girls					
	Additive genetic factors			Unique environment			Additive genetic factors			Unique environment		
	a^2	95% CI		e^2	95% CI		a^2	95% CI		e^2	95% CI	
		LL	UL		LL	UL		LL	UL		LL	UL
**Weight measures**												
Weight	0.94	0.90	0.96	0.06	0.04	0.10	0.83	0.71	0.90	0.17	0.10	0.29
BMI	0.94	0.89	0.96	0.06	0.04	0.11	0.84	0.73	0.90	0.16	0.10	0.27
**Skinfolds**												
Triceps	0.87	0.78	0.92	0.13	0.08	0.22	0.81	0.70	0.88	0.19	0.12	0.30
Biceps	0.84	0.74	0.90	0.16	0.10	0.26	0.68	0.52	0.79	0.32	0.21	0.48
Subscapular	0.85	0.75	0.91	0.15	0.09	0.25	0.88	0.81	0.93	0.12	0.07	0.19
Suprailiac	0.86	0.76	0.91	0.14	0.09	0.24	0.82	0.70	0.89	0.18	0.11	0.30
Calf	0.83	0.72	0.89	0.17	0.11	0.28	0.74	0.61	0.83	0.26	0.17	0.39
Front thigh	0.89	0.83	0.93	0.11	0.07	0.17	0.80	0.68	0.88	0.20	0.12	0.32
Abdominal	0.87	0.78	0.92	0.13	0.08	0.22	0.78	0.63	0.86	0.22	0.14	0.37
**Circumferences**												
Waist	0.87	0.79	0.92	0.13	0.08	0.21	0.78	0.62	0.86	0.22	0.14	0.38
Hip	0.93	0.89	0.96	0.07	0.04	0.11	0.85	0.73	0.91	0.15	0.09	0.27
Upper arm	0.76	0.62	0.85	0.24	0.15	0.38	0.71	0.55	0.81	0.29	0.19	0.45
Upper arm flexed	0.77	0.63	0.85	0.23	0.15	0.37	0.57	0.37	0.71	0.43	0.29	0.63
Forearm	0.55	0.35	0.69	0.45	0.31	0.65	0.78	0.64	0.86	0.22	0.14	0.36
Thigh	0.91	0.86	0.94	0.09	0.06	0.14	0.59	0.39	0.73	0.41	0.27	0.61
Calf	0.78	0.67	0.86	0.22	0.14	0.33	0.83	0.71	0.90	0.17	0.10	0.29
**Diameters**												
Biacromial	0.89	0.81	0.93	0.11	0.07	0.19	0.75	0.59	0.84	0.25	0.16	0.41
Bicristal	0.90	0.84	0.94	0.10	0.06	0.16	0.61	0.14	0.76	0.39	0.24	0.86
Humerus	0.87	0.78	0.91	0.13	0.09	0.22	0.73	0.56	0.83	0.27	0.17	0.44
Femur	0.88	0.80	0.92	0.12	0.08	0.20	0.24	0.01	0.46	0.76	0.54	0.99
**Height measures**												
Height	0.95	0.92	0.97	0.05	0.03	0.08	0.87	0.79	0.92	0.13	0.08	0.21
Sitting height	0.94	0.91	0.96	0.06	0.04	0.09	0.86	0.77	0.91	0.14	0.09	0.23
**Factors**												
First factor	0.89	0.82	0.93	0.11	0.07	0.18	0.86	0.76	0.91	0.14	0.09	0.24
Second factor	0.95	0.92	0.97	0.05	0.03	0.08	0.87	0.79	0.92	0.13	0.08	0.21

Supplementary table 6. Additive genetic correlations (right triangular matrix) and unique environmental correlations (left triangular matrix) of anthropometric traits with 95% confidence intervals in boys and girls.

	**1**	**2**	**3**	**4**	**5**	**6**	**7**	**8**	**9**	**10**	**11**	**12**	**13**	**14**	**15**	**16**	**17**	**18**	**19**	**20**	**21**	**22**
**1**		0.90 0.88, 0.92	0.78 0.71, 0.83	0.72 0.64, 0.79	0.80 0.75, 0.85	0.80 0.75, 0.85	0.77 0.70, 0.83	0.77 0.71, 0.82	0.78 0.71, 0.83	0.93 0.91, 0.95	0.96 0.95, 0.97	0.92 0.89, 0.94	0.92 0.89, 0.95	0.95 0.92, 0.97	0.94 0.92, 0.96	0.91 0.87, 0.95	0.75 0.68, 0.80	0.84 0.78, 0.88	0.76 0.69, 0.82	0.84 0.78, 0.89	0.68 0.61, 0.74	0.66 0.59, 0.73
**2**	0.93 0.90, 0.95		0.85 0.81, 0.89	0.82 0.76, 0.87	0.87 0.83, 0.90	0.86 0.82, 0.90	0.82 0.76, 0.87	0.84 0.79, 0.88	0.84 0.80, 0.88	0.95 0.94, 0.97	0.90 0.88, 0.92	0.95 0.93, 0.97	0.95 0.92, 0.98	0.92 0.88, 0.95	0.93 0.90, 0.95	0.87 0.83, 0.91	0.53 0.42, 0.61	0.64 0.55, 0.72	0.58 0.48, 0.66	0.76 0.67, 0.83	0.30 0.19, 0.41	0.36 0.25, 0.46
**3**	0.55 0.39, 0.68	0.59 0.43, 0.71		0.92 0.89, 0.95	0.88 0.84, 0.91	0.92 0.88, 0.95	0.91 0.87, 0.94	0.93 0.89, 0.96	0.92 0.89, 0.95	0.85 0.80, 0.90	0.80 0.75, 0.85	0.87 0.82, 0.91	0.86 0.80, 0.92	0.80 0.72, 0.87	0.83 0.76, 0.88	0.73 0.65, 0.80	0.41 0.29, 0.52	0.61 0.51, 0.70	0.42 0.30, 0.53	0.63 0.52, 0.73	0.27 0.15, 0.38	0.27 0.15, 0.38
**4**	0.52 0.35, 0.66	0.53 0.36, 0.66	0.63 0.49, 0.73		0.86 0.81, 0.91	0.86 0.81, 0.90	0.88 0.83, 0.92	0.85 0.80, 0.90	0.85 0.79, 0.89	0.81 0.74, 0.86	0.76 0.68, 0.82	0.83 0.77, 0.89	0.82 0.73, 0.89	0.72 0.61, 0.81	0.77 0.68, 0.84	0.67 0.57, 0.75	0.35 0.21, 0.47	0.52 0.40, 0.62	0.36 0.23, 0.48	0.61 0.49, 0.72	0.19 0.06, 0.31	0.21 0.08, 0.33
**5**	0.63 0.49, 0.74	0.71 0.58, 0.80	0.62 0.48, 0.74	0.53 0.36, 0.66		0.92 0.89, 0.95	0.81 0.75, 0.86	0.84 0.79, 0.88	0.92 0.88, 0.94	0.88 0.84, 0.91	0.81 0.76, 0.85	0.86 0.81, 0.90	0.86 0.80, 0.91	0.79 0.70, 0.86	0.81 0.75, 0.86	0.70 0.62, 0.77	0.46 0.34, 0.56	0.63 0.53, 0.71	0.46 0.35, 0.57	0.59 0.48, 0.69	0.31 0.19, 0.41	0.32 0.21, 0.43
**6**	0.71 0.58, 0.80	0.74 0.63, 0.83	0.63 0.49, 0.74	0.63 0.49, 0.74	0.69 0.57, 0.78		0.88 0.83, 0.92	0.88 0.84, 0.92	0.98 0.96, 0.99	0.87 0.82, 0.91	0.83 0.77, 0.87	0.87 0.82, 0.91	0.87 0.81, 0.92	0.81 0.73, 0.88	0.82 0.76, 0.87	0.73 0.65, 0.81	0.43 0.31, 0.54	0.63 0.53, 0.71	0.51 0.39, 0.61	0.60 0.48, 0.71	0.31 0.20, 0.42	0.32 0.20, 0.43
**7**	0.46 0.28, 0.61	0.52 0.35, 0.65	0.62 0.48, 0.72	0.62 0.49, 0.73	0.55 0.40, 0.68	0.57 0.42, 0.69		0.94 0.91, 0.97	0.85 0.80, 0.89	0.80 0.73, 0.85	0.80 0.74, 0.85	0.81 0.74, 0.87	0.80 0.72, 0.88	0.77 0.67, 0.85	0.81 0.74, 0.87	0.71 0.62, 0.79	0.43 0.30, 0.54	0.61 0.51, 0.71	0.42 0.29, 0.54	0.61 0.48, 0.72	0.30 0.18, 0.41	0.29 0.17, 0.41
**8**	0.46 0.28, 0.60	0.48 0.31, 0.63	0.50 0.34, 0.63	0.57 0.41, 0.69	0.53 0.37, 0.67	0.54 0.39, 0.67	0.54 0.39, 0.66		0.86 0.82, 0.90	0.83 0.78, 0.88	0.80 0.74, 0.84	0.83 0.76, 0.88	0.82 0.74, 0.88	0.76 0.67, 0.83	0.82 0.76, 0.87	0.70 0.62, 0.78	0.45 0.34, 0.56	0.59 0.49, 0.68	0.38 0.25, 0.49	0.60 0.48, 0.70	0.29 0.17, 0.40	0.30 0.18, 0.41
**9**	0.69 0.55, 0.79	0.74 0.62, 0.82	0.64 0.50, 0.75	0.63 0.49, 0.74	0.62 0.48, 0.73	0.77 0.68, 0.84	0.64 0.51, 0.74	0.66 0.53, 0.76		0.86 0.82, 0.90	0.80 0.74, 0.84	0.83 0.77, 0.88	0.84 0.77, 0.90	0.77 0.68, 0.84	0.83 0.76, 0.88	0.71 0.63, 0.79	0.41 0.28, 0.52	0.62 0.52, 0.71	0.43 0.30, 0.54	0.58 0.46, 0.69	0.28 0.16, 0.39	0.30 0.18, 0.41
**10**	0.74 0.63, 0.82	0.76 0.66, 0.83	0.51 0.35, 0.65	0.44 0.27, 0.59	0.55 0.40, 0.68	0.58 0.43, 0.70	0.44 0.27, 0.59	0.31 0.12, 0.48	0.57 0.41, 0.69		0.93 0.90, 0.95	0.95 0.92, 0.97	0.94 0.90, 0.97	0.94 0.90, 0.97	0.95 0.92, 0.98	0.86 0.81, 0.91	0.60 0.50, 0.68	0.72 0.64, 0.78	0.64 0.54, 0.73	0.78 0.69, 0.85	0.45 0.34, 0.55	0.45 0.34, 0.54
**11**	0.90 0.85, 0.93	0.86 0.79, 0.91	0.59 0.44, 0.71	0.48 0.31, 0.63	0.64 0.49, 0.75	0.64 0.50, 0.75	0.44 0.27, 0.60	0.55 0.39, 0.68	0.70 0.57, 0.80	0.68 0.55, 0.78		0.93 0.90, 0.95	0.93 0.89, 0.96	0.96 0.93, 0.98	0.96 0.94, 0.98	0.88 0.84, 0.92	0.70 0.63, 0.77	0.81 0.75, 0.86	0.69 0.61, 0.76	0.80 0.73, 0.86	0.59 0.50, 0.66	0.58 0.50, 0.66
**12**	0.68 0.57, 0.77	0.66 0.55, 0.75	0.56 0.41, 0.68	0.33 0.16, 0.49	0.47 0.31, 0.61	0.50 0.34, 0.63	0.39 0.22, 0.54	0.41 0.24, 0.56	0.56 0.41, 0.68	0.60 0.47, 0.71	0.69 0.58, 0.77		1.00 0.99, NA	0.94 0.92, 0.96	0.94 0.91, 0.97	0.89 0.84, 0.94	0.64 0.54, 0.72	0.70 0.61, 0.78	0.68 0.59, 0.76	0.69 0.59, 0.78	0.41 0.29, 0.52	0.45 0.33, 0.55
**13**	0.58 0.45, 0.69	0.57 0.43, 0.68	0.44 0.28, 0.58	0.23 0.05, 0.40	0.41 0.24, 0.56	0.39 0.22, 0.54	0.27 0.09, 0.44	0.32 0.14, 0.48	0.42 0.25, 0.57	0.52 0.37, 0.64	0.57 0.44, 0.69	0.86 0.81, 0.90		0.96 0.93, 0.98	0.95 0.90, 0.98	0.90 0.84, 0.95	0.68 0.58, 0.77	0.70 0.60, 0.78	0.66 0.56, 0.74	0.70 0.59, 0.79	0.43 0.31, 0.54	0.48 0.36, 0.58

**14**	0.62 0.49, 0.72	0.56 0.42, 0.67	0.38 0.21, 0.53	0.35 0.17, 0.51	0.36 0.19, 0.52	0.40 0.22, 0.55	0.28 0.10, 0.45	0.31 0.12, 0.47	0.44 0.27, 0.59	0.51 0.37, 0.64	0.59 0.46, 0.69	0.90 0.85, 0.93	0.82 0.75, 0.87		0.94 0.90, 0.98	0.94 0.89, 0.99	0.74 0.65, 0.82	0.81 0.73, 0.87	0.83 0.76, 0.90	0.73 0.62, 0.81	0.58 0.47, 0.67	0.62 0.52, 0.71
**15**	0.80 0.71, 0.86	0.80 0.72, 0.86	0.51 0.35, 0.64	0.45 0.28, 0.59	0.61 0.46, 0.72	0.64 0.50, 0.75	0.46 0.29, 0.60	0.44 0.27, 0.58	0.55 0.39, 0.67	0.60 0.46, 0.71	0.74 0.65, 0.82	0.63 0.51, 0.73	0.54 0.40, 0.66	0.57 0.43, 0.68		0.94 0.91, 0.98	0.67 0.57, 0.75	0.76 0.67, 0.83	0.64 0.54, 0.73	0.78 0.70, 0.85	0.49 0.39, 0.59	0.52 0.41, 0.61
**16**	0.48 0.31, 0.63	0.48 0.31, 0.62	0.30 0.10, 0.47	0.30 0.11, 0.48	0.32 0.12, 0.49	0.38 0.18, 0.55	0.28 0.08, 0.45	0.31 0.11, 0.48	0.35 0.15, 0.52	0.44 0.26, 0.59	0.57 0.41, 0.70	0.39 0.22, 0.54	0.34 0.17, 0.50	0.31 0.14, 0.47	0.46 0.30, 0.60		0.68 0.58, 0.77	0.77 0.69, 0.85	0.69 0.60, 0.78	0.80 0.71, 0.88	0.49 0.38, 0.58	0.51 0.41, 0.60
**17**	0.63 0.48, 0.74	0.56 0.40, 0.69	0.37 0.17, 0.54	0.33 0.13, 0.50	0.38 0.18, 0.55	0.49 0.30, 0.64	0.30 0.09, 0.48	0.28 0.07, 0.46	0.43 0.23, 0.59	0.50 0.33, 0.64	0.57 0.40, 0.69	0.46 0.29, 0.61	0.36 0.18, 0.52	0.45 0.28, 0.59	0.46 0.28, 0.60	0.27 0.07, 0.45		0.70 0.61, 0.77	0.73 0.65, 0.80	0.64 0.53, 0.74	0.74 0.68, 0.80	0.70 0.62, 0.77
**18**	0.56 0.40, 0.69	0.53 0.36, 0.66	0.30 0.11, 0.48	0.31 0.11, 0.48	0.24 0.04, 0.42	0.43 0.24, 0.58	0.18 -0.01, 0.37	0.11 -0.09, 0.31	0.28 0.08, 0.46	0.60 0.45, 0.72	0.46 0.29, 0.61	0.42 0.25, 0.57	0.45 0.28, 0.59	0.44 0.28, 0.58	0.41 0.23, 0.56	0.22 0.02, 0.41	0.42 0.24, 0.58		0.70 0.61, 0.78	0.75 0.66, 0.83	0.76 0.69, 0.81	0.69 0.61, 0.76
**19**	0.40 0.21, 0.56	0.37 0.17, 0.54	0.30 0.10, 0.48	0.33 0.13, 0.50	0.21 -0.01, 0.40	0.23 0.02, 0.42	0.12 -0.08, 0.32	0.21 0.00, 0.40	0.35 0.15, 0.52	0.24 0.04, 0.43	0.37 0.17, 0.53	0.36 0.18, 0.53	0.48 0.31, 0.62	0.39 0.22, 0.54	0.36 0.18, 0.53	0.15 -0.05, 0.34	0.23 0.03, 0.41	0.24 0.05, 0.43		0.74 0.64, 0.82	0.70 0.62, 0.76	0.68 0.60, 0.75
**20**	0.48 0.33, 0.62	0.41 0.24, 0.56	0.20 0.01, 0.38	0.17 -0.03, 0.35	0.24 0.05, 0.42	0.30 0.11, 0.47	0.17 -0.02, 0.35	0.24 0.05, 0.42	0.25 0.06, 0.43	0.38 0.20, 0.53	0.46 0.29, 0.60	0.50 0.34, 0.63	0.41 0.24, 0.55	0.49 0.34, 0.62	0.45 0.29, 0.58	0.40 0.23, 0.55	0.31 0.12, 0.47	0.38 0.21, 0.53	0.21 0.02, 0.38		0.59 0.48, 0.68	0.59 0.48, 0.68
**21**	0.73 0.61, 0.81	0.43 0.24, 0.59	0.25 0.05, 0.43	0.28 0.08, 0.46	0.21 0.01, 0.41	0.37 0.17, 0.54	0.19 -0.01, 0.38	0.20 -0.00, 0.40	0.32 0.11, 0.50	0.39 0.20, 0.56	0.62 0.48, 0.74	0.47 0.30, 0.62	0.40 0.22, 0.56	0.46 0.29, 0.60	0.50 0.33, 0.64	0.42 0.23, 0.58	0.54 0.37, 0.67	0.46 0.28, 0.60	0.34 0.15, 0.51	0.43 0.25, 0.58		0.87 0.84, 0.90
**22**	0.59 0.43, 0.71	0.37 0.18, 0.54	0.18 -0.03, 0.37	0.11 -0.10, 0.31	0.14 -0.07, 0.34	0.19 0.02, 0.39	0.14 -0.07, 0.33	0.17 -0.04, 0.36	0.19 -0.02, 0.39	0.31 0.11, 0.49	0.53 0.36, 0.66	0.36 0.17, 0.52	0.34 0.15, 0.50	0.31 0.12, 0.48	0.38 0.20, 0.54	0.32 0.13, 0.50	0.42 0.23, 0.58	0.44 0.26, 0.59	0.24 0.04, 0.43	0.30 0.12, 0.47	0.76 0.66, 0.84	

Note: 1=weight, 2=bmi, 3=tricepsSF, 4=bicepsSF, 5=subscapularSF, 6=suprailiacSF, 7=calfSF, 8=frontthighSF, 9=abdominalSF, 10=waistC, 11=hipC, 12=upperarmC, 13=upper armflexedC, 14=forearmC, 15=thighC, 16=calfC, 17=biacromialD, 18=bicristalD, 19=humerusD, 20=femurD, 21=height, 22=sitting height (SF=skinfold; C=circumference; D=diameter)

Supplementary table 7. Additive genetic correlations in boys (right triangular matrix) and girls (left triangular matrix) with 95% confidence intervals.

	**1**	**2**	**3**	**4**	**5**	**6**	**7**	**8**	**9**	**10**	**11**	**12**	**13**	**14**	**15**	**16**	**17**	**18**	**19**	**20**	**21**	**22**
**1**		0.90<br>0.86,<br>0.93	0.72<br>0.63,<br>0.80	0.69<br>0.58,<br>0.77	0.81<br>0.73,<br>0.86	0.79<br>0.71,<br>0.84	0.70<br>0.60,<br>0.79	0.71<br>0.62,<br>0.79	0.77<br>0.69,<br>0.83	0.93<br>0.90,<br>0.96	0.96<br>0.94,<br>0.98	0.92<br>0.88,<br>0.95	0.89<br>0.84,<br>0.94	0.96<br>0.92,<br>0.99	0.89<br>0.85,<br>0.92	0.97<br>0.93,<br>0.99	0.72<br>0.62,<br>0.79	0.82<br>0.75,<br>0.87	0.83<br>0.76,<br>0.89	0.79<br>0.71,<br>0.85	0.68<br>0.59,<br>0.76	0.67<br>0.57,<br>0.75
**2**	0.90<br>0.86,<br>0.93		0.83<br>0.77,<br>0.88	0.81<br>0.73,<br>0.87	0.89<br>0.85,<br>0.92	0.88<br>0.83,<br>0.92	0.83<br>0.76,<br>0.89	0.83<br>0.77,<br>0.88	0.88<br>0.83,<br>0.92	0.96<br>0.93,<br>0.97	0.93<br>0.90,<br>0.95	0.97<br>0.95,<br>0.99	0.95<br>0.91,<br>0.98	0.95<br>0.90,<br>0.99	0.91<br>0.88,<br>0.94	0.93<br>0.88,<br>0.97	0.47<br>0.33,<br>0.59	0.61<br>0.49,<br>0.70	0.61<br>0.48,<br>0.71	0.69<br>0.58,<br>0.77	0.30<br>0.15,<br>0.44	0.36<br>0.22,<br>0.49
**3**	0.84<br>0.76,<br>0.90	0.87<br>0.80,<br>0.93		0.92<br>0.89,<br>0.95	0.87<br>0.81,<br>0.91	0.94<br>0.90,<br>0.96	0.93<br>0.89,<br>0.96	0.94<br>0.91,<br>0.97	0.94<br>0.91,<br>0.96	0.82<br>0.75,<br>0.88	0.78<br>0.70,<br>0.84	0.87<br>0.79,<br>0.94	0.81<br>0.71,<br>0.90	0.81<br>0.68,<br>0.94	0.75<br>0.66,<br>0.82	0.75<br>0.63,<br>0.86	0.32<br>0.16,<br>0.47	0.57<br>0.44,<br>0.68	0.39<br>0.22,<br>0.54	0.52<br>0.37,<br>0.64	0.20<br>0.04,<br>0.35	0.19<br>0.03,<br>0.34
**4**	0.76<br>0.64,<br>0.86	0.84<br>0.74,<br>0.92	0.93<br>0.86,<br>1.00		0.86<br>0.79,<br>0.92	0.88<br>0.82,<br>0.93	0.91<br>0.85,<br>0.96	0.86<br>0.80,<br>0.91	0.87<br>0.81,<br>0.92	0.80<br>0.71,<br>0.87	0.75<br>0.66,<br>0.83	0.83<br>0.73,<br>0.91	0.76<br>0.63,<br>0.88	0.76<br>0.60,<br>0.91	0.72<br>0.62,<br>0.80	0.69<br>0.56,<br>0.81	0.26<br>0.09,<br>0.42	0.52<br>0.38,<br>0.64	0.33<br>0.15,<br>0.48	0.51<br>0.36,<br>0.64	0.17<br>0.01,<br>0.33	0.15<br>-.01,<br>0.31
**5**	0.81<br>0.73,<br>0.87	0.85<br>0.79,<br>0.90	0.89<br>0.83,<br>0.94	0.87<br>0.78,<br>0.94		0.92<br>0.88,<br>0.94	0.82<br>0.74,<br>0.89	0.86<br>0.80,<br>0.91	0.91<br>0.87,<br>0.95	0.89<br>0.83,<br>0.94	0.84<br>0.77,<br>0.89	0.92<br>0.86,<br>0.97	0.88<br>0.79,<br>0.95	0.87<br>0.75,<br>0.97	0.79<br>0.71,<br>0.85	0.76<br>0.65,<br>0.85	0.39<br>0.23,<br>0.54	0.57<br>0.44,<br>0.68	0.48<br>0.32,<br>0.61	0.56<br>0.41,<br>0.67	0.28<br>0.12,<br>0.42	0.28<br>0.12,<br>0.43
**6**	0.84<br>0.76,<br>0.91	0.86<br>0.77,<br>0.91	0.90<br>0.83,<br>0.95	0.84<br>0.75,<br>0.91	0.94<br>0.90,<br>0.98		0.88<br>0.82,<br>0.93	0.90<br>0.85,<br>0.94	0.98<br>0.97,<br>0.99	0.86<br>0.80,<br>0.91	0.83<br>0.77,<br>0.88	0.89<br>0.82,<br>0.95	0.86<br>0.77,<br>0.93	0.92<br>0.80,<br>1.00	0.79<br>0.70,<br>0.85	0.77<br>0.65,<br>0.86	0.40<br>0.24,<br>0.55	0.62<br>0.49,<br>0.72	0.52<br>0.37,<br>0.65	0.55<br>0.40,<br>0.66	0.29<br>0.13,<br>0.43	0.26<br>0.10,<br>0.41
**7**	0.85<br>0.76,<br>0.92	0.79<br>0.70,<br>0.87	0.88<br>0.82,<br>0.93	0.84<br>0.75,<br>0.91	0.80<br>0.71,<br>0.87	0.87<br>0.79,<br>0.93		0.92<br>0.87,<br>0.95	0.87<br>0.81,<br>0.92	0.81<br>0.72,<br>0.88	0.75<br>0.65,<br>0.83	0.83<br>0.72,<br>0.93	0.75<br>0.62,<br>0.87	0.81<br>0.64,<br>0.96	0.73<br>0.63,<br>0.82	0.71<br>0.57,<br>0.82	0.28<br>0.11,<br>0.44	0.52<br>0.37,<br>0.65	0.33<br>0.15,<br>0.49	0.49<br>0.33,<br>0.62	0.17<br>0.00,<br>0.32	0.15<br>0.01,<br>0.31
**8**	0.85<br>0.77,<br>0.91	0.83<br>0.75,<br>0.90	0.90<br>0.83,<br>0.96	0.84<br>0.74,<br>0.93	0.82<br>0.75,<br>0.89	0.86<br>0.79,<br>0.91	0.97<br>0.92,<br>1.00		0.88<br>0.83,<br>0.92	0.79<br>0.71,<br>0.85	0.76<br>0.68,<br>0.82	0.83<br>0.74,<br>0.90	0.77<br>0.66,<br>0.86	0.75<br>0.60,<br>0.88	0.75<br>0.67,<br>0.82	0.70<br>0.58,<br>0.80	0.35<br>0.19,<br>0.49	0.51<br>0.37,<br>0.62	0.31<br>0.14,<br>0.46	0.51<br>0.37,<br>0.63	0.20<br>0.04,<br>0.35	0.19<br>0.03,<br>0.33
**9**	0.80<br>0.69,<br>0.88	0.82<br>0.71,<br>0.89	0.91<br>0.84,<br>0.96	0.83<br>0.72,<br>0.92	0.92<br>0.87,<br>0.97	0.97<br>0.95,<br>0.99	0.82<br>0.73,<br>0.89	0.84<br>0.76,<br>0.89		0.86<br>0.80,<br>0.91	0.82<br>0.75,<br>0.87	0.89<br>0.82,<br>0.94	0.84<br>0.75,<br>0.92	0.91<br>0.79,<br>NA	0.79<br>0.71,<br>0.85	0.74<br>0.63,<br>0.84	0.35<br>0.18,<br>0.49	0.59<br>0.46,<br>0.69	0.45<br>0.29,<br>0.59	0.52<br>0.38,<br>0.64	0.24<br>0.08,<br>0.39	0.25<br>0.09,<br>0.39
**10**	0.93<br>0.89,<br>0.96	0.95<br>0.92,<br>0.98	0.88<br>0.81,<br>0.94	0.81<br>0.69,<br>0.89	0.92<br>0.83,<br>1.00	0.88<br>0.81,<br>0.95	0.79<br>0.68,<br>0.88	0.88<br>0.80,<br>0.96	0.87<br>0.79,<br>0.95		0.95<br>0.91,<br>0.97	0.96<br>0.93,<br>0.99	0.92<br>0.86,<br>0.97	0.97<br>0.91,<br>1.00	0.94<br>0.89,<br>0.98	0.91<br>0.84,<br>0.96	0.55<br>0.41,<br>0.66	0.71<br>0.60,<br>0.79	0.70<br>0.59,<br>0.80	0.71<br>0.60,<br>0.80	0.45<br>0.30,<br>0.57	0.44<br>0.29,<br>0.57
**11**	0.96<br>0.94,<br>0.97	0.89<br>0.83,<br>0.92	0.83<br>0.75,<br>0.89	0.76<br>0.64,<br>0.85	0.78<br>0.70,<br>0.85	0.82<br>0.73,<br>0.89	0.85<br>0.77,<br>0.92	0.84<br>0.76,<br>0.90	0.76<br>0.66,<br>0.84	0.91<br>0.85,<br>0.94		0.94<br>0.90,<br>0.97	0.90<br>0.84,<br>0.95	0.97<br>0.93,<br>1.00	0.93<br>0.90,<br>0.96	0.94<br>0.88,<br>0.98	0.63<br>0.52,<br>0.72	0.77<br>0.68,<br>0.84	0.76<br>0.67,<br>0.84	0.74<br>0.64,<br>0.81	0.57<br>0.45,<br>0.66	0.56<br>0.44,<br>0.66
**12**	0.92<br>0.87,<br>0.95	0.93<br>0.89,<br>0.96	0.87<br>0.80,<br>0.92	0.83<br>0.73,<br>0.92	0.81<br>0.72,<br>0.87	0.84<br>0.76,<br>0.91	0.79<br>0.69,<br>0.87	0.82<br>0.73,<br>0.89	0.76<br>0.65,<br>0.85	0.93<br>0.87,<br>0.96	0.92<br>0.87,<br>0.95		0.98<br>0.95,<br>1.00	0.95<br>0.91,<br>0.98	0.94<br>0.90,<br>0.99	0.91<br>0.85,<br>0.97	0.59<br>0.44,<br>0.71	0.66<br>0.53,<br>0.77	0.68<br>0.55,<br>0.78	0.60<br>0.46,<br>0.71	0.38<br>0.23,<br>0.52	0.44<br>0.29,<br>0.58
**13**	0.95<br>0.91,<br>0.99	0.95<br>0.91,<br>0.99	0.90<br>0.83,<br>0.96	0.84<br>0.72,<br>0.94	0.85<br>0.76,<br>0.92	0.87<br>0.78,<br>0.94	0.83<br>0.72,<br>0.92	0.85<br>0.75,<br>0.93	0.83<br>0.72,<br>0.93	0.95<br>0.90,<br>0.99	0.95<br>0.90,<br>0.98	1.00<br>0.99,<br>NA		0.95<br>0.90,<br>0.98	0.93<br>0.86,<br>1.00	0.91<br>0.83,<br>0.97	0.55<br>0.40,<br>0.68	0.63<br>0.49,<br>0.73	0.66<br>0.53,<br>0.78	0.55<br>0.40,<br>0.67	0.37<br>0.22,<br>0.51	0.45<br>0.30,<br>0.58

**14**	0.91 0.85, 0.94	0.84 0.75, 0.89	0.78 0.69, 0.85	0.67 0.54, 0.78	0.68 0.57, 0.77	0.73 0.62, 0.82	0.74 0.63, 0.82	0.76 0.66, 0.84	0.63 0.49, 0.73	0.87 0.81, 0.92	0.91 0.86, 0.94	0.94 0.91, 0.96	0.97 0.93, 1.00		0.97 0.91, 1.00	0.96 0.90, 1.00	0.78 0.64, 0.89	0.81 0.70, 0.91	0.92 0.83, 1.00	0.66 0.51, 0.77	0.59 0.44, 0.71	0.69 0.53, 0.82
**15**	0.97 0.94, 0.99	0.93 0.89, 0.96	0.89 0.81, 0.96	0.79 0.65, 0.90	0.83 0.73, 0.90	0.86 0.77, 0.93	0.87 0.77, 0.95	0.87 0.78, 0.94	0.87 0.76, 0.95	0.95 0.90, 0.99	0.98 0.96, 1.00	0.94 0.88, 0.98	0.98 0.92, NA	0.93 0.87, 0.97		0.96 0.91, 1.00	0.55 0.41, 0.66	0.62 0.50, 0.72	0.63 0.51, 0.73	0.73 0.64, 0.81	0.45 0.31, 0.57	0.48 0.34, 0.59
**16**	0.80 0.70, 0.86	0.80 0.70, 0.86	0.69 0.57, 0.79	0.63 0.47, 0.75	0.62 0.50, 0.72	0.67 0.55, 0.77	0.71 0.58, 0.82	0.71 0.59, 0.81	0.66 0.53, 0.78	0.79 0.72, 0.86	0.80 0.72, 0.86	0.84 0.76, 0.91	0.88 0.79, 0.95	0.83 0.72, 0.89	0.93 0.87, 0.96		0.73 0.59, 0.86	0.84 0.74, 0.94	0.76 0.64, 0.85	0.78 0.66, 0.87	0.57 0.44, 0.69	0.58 0.45, 0.69
**17**	0.80 0.69, 0.87	0.58 0.43, 0.70	0.51 0.34, 0.66	0.44 0.24, 0.61	0.48 0.30, 0.63	0.45 0.27, 0.60	0.61 0.44, 0.76	0.58 0.42, 0.72	0.49 0.30, 0.64	0.65 0.50, 0.76	0.78 0.68, 0.85	0.73 0.56, 0.84	0.87 0.70, NA	0.74 0.61, 0.84	0.77 0.64, 0.87	0.67 0.52, 0.79		0.69 0.57, 0.78	0.83 0.75, 0.89	0.53 0.39, 0.65	0.75 0.67, 0.82	0.71 0.62, 0.79
**18**	0.86 0.76, 0.93	0.68 0.54, 0.79	0.64 0.49, 0.77	0.53 0.33, 0.70	0.69 0.55, 0.81	0.67 0.51, 0.80	0.72 0.57, 0.85	0.70 0.55, 0.83	0.68 0.51, 0.83	0.73 NA, 0.82	0.84 0.75, 0.92	0.74 0.65, 0.85	0.75 0.60, 0.86	0.80 0.69, 0.88	0.92 0.78, NA	0.67 0.50, 0.80	0.67 0.46, 0.79		0.74 0.64, 0.83	0.76 0.67, 0.84	0.76 0.68, 0.83	0.68 0.58, 0.77
**19**	0.68 0.55, 0.79	0.55 0.38, 0.69	0.46 0.28, 0.61	0.43 0.22, 0.61	0.46 0.29, 0.62	0.51 0.32, 0.68	0.54 0.36, 0.71	0.48 0.29, 0.64	0.41 0.21, 0.59	0.62 0.45, 0.79	0.62 0.45, 0.77	0.69 0.55, 0.81	0.65 0.48, 0.78	0.72 0.56, 0.83	0.65 0.47, 0.80	0.58 0.41, 0.72	0.59 0.40, 0.74	0.62 0.44, 0.76		0.68 0.56, 0.77	0.76 0.67, 0.83	0.75 0.66, 0.83
**20**	0.91 0.80, 1.00	0.90 0.75, 1.00	0.88 0.65, 1.00	0.82 0.57, 1.00	0.75 0.53, 1.00	0.81 0.57, 1.00	0.84 0.61, 1.00	0.81 0.59, 1.00	0.82 0.54, NA	0.91 0.76, 1.00	0.92 0.79, 1.00	0.90 0.76, 1.00	0.95 0.79, NA	0.93 0.80, 1.00	0.87 0.65, NA	0.88 0.73, 1.00	0.77 0.54, 0.97	0.72 0.43, 1.00	0.88 0.67, 1.00		0.58 0.46, 0.68	0.58 0.46, 0.69
**21**	0.67 0.55, 0.75	0.28 0.10, 0.43	0.34 0.17, 0.49	0.20 0.01, 0.38	0.34 0.18, 0.48	0.35 0.18, 0.49	0.46 0.30, 0.61	0.40 0.24, 0.55	0.31 0.14, 0.47	0.44 0.27, 0.58	0.60 0.48, 0.70	0.44 0.27, 0.58	0.48 0.30, 0.63	0.56 0.42, 0.68	0.52 0.35, 0.66	0.38 0.21, 0.52	0.72 0.60, 0.81	0.73 0.62, 0.82	0.61 0.47, 0.72	0.55 0.32, 0.73		0.87 0.83, 0.91
**22**	0.65 0.53, 0.74	0.33 0.15, 0.48	0.38 0.21, 0.52	0.28 0.09, 0.46	0.38 0.22, 0.52	0.41 0.24, 0.55	0.50 0.34, 0.64	0.47 0.31, 0.60	0.36 0.19, 0.52	0.40 0.21, 0.56	0.61 0.48, 0.71	0.44 0.27, 0.58	0.49 0.31, 0.64	0.55 0.38, 0.68	0.54 0.37, 0.68	0.40 0.23, 0.54	0.68 0.55, 0.78	0.68 0.53, 0.78	0.56 0.40, 0.68	0.54 0.30, 0.73	0.88 0.83, 0.91	

Note: 1=weight, 2=bmi, 3=tricepsSF, 4=bicepsSF, 5=subscapularSF, 6=suprailiacSF, 7=calfSF, 8=frontthighSF, 9=abdominalSF, 10=waistC, 11=hipC, 12=upperarmC, 13=upper armflexedC, 14=forearmC, 15=thighC, 16=calfC, 17=biacromialD, 18=bicristalD, 19=humerusD, 20=femurD, 21=height, 22=sitting height (SF=skinfold; C=circumference; D=diameter)

Supplementary table 8. Unique environmental correlations in boys (right triangular matrix) and girls (left triangular matrix) of anthropometric traits with 95% confidence intervals.

	**1**	**2**	**3**	**4**	**5**	**6**	**7**	**8**	**9**	**10**	**11**	**12**	**13**	**14**	**15**	**16**	**17**	**18**	**19**	**20**	**21**	**22**
**1**		0.92 0.86, 0.96	0.62 0.39, 0.77	0.53 0.28, 0.71	0.73 0.56, 0.85	0.75 0.59, 0.86	0.49 0.23, 0.69	0.59 0.36, 0.75	0.72 0.55, 0.84	0.73 0.56, 0.84	0.76 0.56, 0.87	0.69 0.53, 0.81	0.66 0.46, 0.79	0.50 0.31, 0.66	0.84 0.72, 0.91	0.17 -0.08, 0.41	0.64 0.43, 0.79	0.57 0.32, 0.74	0.22 -0.07, 0.48	0.51 0.25, 0.70	0.67 0.47, 0.80	0.49 0.23, 0.68
**2**	0.94 0.89, 0.97		0.70 0.51, 0.83	0.61 0.38, 0.77	0.80 0.66, 0.89	0.79 0.64, 0.88	0.46 0.19, 0.66	0.59 0.36, 0.75	0.77 0.61, 0.87	0.74 0.59, 0.85	0.78 0.64, 0.88	0.63 0.45, 0.76	0.55 0.32, 0.72	0.40 0.18, 0.58	0.80 0.65, 0.89	0.16 -0.10, 0.41	0.46 0.19, 0.67	0.54 0.29, 0.72	0.24 -0.05, 0.50	0.37 0.10, 0.60	0.33 0.04, 0.57	0.27 - 0.02, 0.52
**3**	0.54 0.30, 0.72	0.54 0.30, 0.72		0.78 0.65, 0.87	0.81 0.68, 0.90	0.67 0.49, 0.81	0.55 0.34, 0.72	0.62 0.40, 0.77	0.75 0.60, 0.85	0.57 0.34, 0.74	0.66 0.45, 0.80	0.44 0.19, 0.65	0.33 0.05, 0.56	0.24 -0.01, 0.48	0.65 0.44, 0.79	0.08 -0.20, 0.35	0.27 -0.03, 0.53	0.30 0.01, 0.54	0.19 -0.10, 0.46	0.29 0.00, 0.54	0.16 -0.14, 0.43	0.08 - 0.22, 0.36
**4**	0.51 0.27, 0.70	0.50 0.27, 0.69	0.51 0.29, 0.69		0.64 0.41, 0.80	0.64 0.42, 0.78	0.51 0.27, 0.69	0.62 0.41, 0.77	0.67 0.48, 0.80	0.46 0.19, 0.66	0.48 0.22, 0.68	0.37 0.10, 0.59	0.22 -0.06, 0.48	0.19 -0.07, 0.44	0.52 0.26, 0.71	0.06 -0.21, 0.34	0.14 -0.17, 0.41	0.41 0.13, 0.63	0.28 -0.01, 0.53	0.10 -0.20, 0.38	0.11 -0.19, 0.39	0.05 - 0.24, 0.34
**5**	0.59 0.36, 0.76	0.67 0.46, 0.81	0.45 0.19, 0.65	0.47 0.22, 0.66		0.81 0.68, 0.89	0.58 0.36, 0.75	0.60 0.38, 0.76	0.72 0.55, 0.84	0.62 0.03, 0.92	0.69 0.49, 0.82	0.43 0.19, 0.63)	0.33 0.07, 0.56	0.24 -0.01, 0.47	0.73 0.55, 0.85	0.14 -0.13, 0.40	0.34 0.03, 0.59	0.45 0.18, 0.66	0.19 -0.11, 0.46	0.25 -0.04, 0.51	0.24 -0.06, 0.50	0.21 - 0.09, 0.48
**6**	0.66 0.42, 0.82	0.78 0.59, 0.89	0.60 0.37, 0.76	0.64 0.43, 0.78	0.55 0.32, 0.73		0.60 0.38, 0.75	0.58 0.36, 0.75	0.83 0.72, 0.90	0.55 0.32, 0.72	0.67 0.46, 0.81	0.49 0.25, 0.67	0.36 0.09, 0.58	0.27 -0.11, 0.51	0.66 0.45, 0.80	0.22 -0.06, 0.47	0.29 -0.02, 0.54	0.40 0.13, 0.63	0.17 -0.13, 0.44	0.33 0.05, 0.57	0.33 0.04, 0.57	0.16 - 0.14, 0.43
**7**	0.46 0.21, 0.66	0.58 0.36, 0.74	0.66 0.49, 0.79	0.70 0.52, 0.81	0.53 0.30, 0.70	0.56 0.34, 0.72		0.63 0.41, 0.78	0.59 0.38, 0.75	0.40 0.13, 0.62	0.40 0.12, 0.62	0.26 -0.02, 0.50	0.21 -0.07, 0.47	0.11 -0.15, 0.36	0.49 0.23, 0.69	0.14 -0.14, 0.40	0.26 -0.05, 0.52	0.14 -0.15, 0.42	0.10 -0.19, 0.38	0.21 -0.08, 0.47	0.28 -0.01, 0.53	0.21 - 0.08, 0.47
**8**	0.40 0.14, 0.62	0.47 0.22, 0.67	0.43 0.19, 0.64	0.52 0.29, 0.70	0.50 0.26, 0.69	0.53 0.31, 0.71	0.50 0.24, 0.68		0.69 0.50, 0.81	0.49 0.24, 0.68	0.56 0.33, 0.73	0.40 0.14, 0.61	0.33 0.05, 0.57	0.23 -0.03, 0.47	0.57 0.33, 0.74	0.27 -0.01, 0.51	0.30 0.01, 0.55	0.31 0.03, 0.55	0.21 -0.08, 0.48	0.19 -0.10, 0.45	0.28 -0.01, 0.53	0.32 0.04, 0.56
**9**	0.64 0.39, 0.80	0.77 0.58, 0.88	0.56 0.33, 0.73	0.59 0.36, 0.75	0.54 0.32, 0.72	0.74 0.59, 0.84	0.67 0.48, 0.80	0.64 0.45, 0.78		0.58 0.36, 0.74	0.69 0.50, 0.82	0.49 0.26, 0.68	0.38 0.11, 0.60	0.27 -0.15, 0.52	0.66 0.45, 0.80	0.23 -0.05, 0.48	0.39 0.10, 0.61	0.33 0.04, 0.56	0.29 0.00, 0.54	0.35 0.06, 0.58	0.31 0.02, 0.55	0.11 - 0.18, 0.39
**10**	0.76 0.61, 0.86	0.77 0.63, 0.87	0.48 NA, 0.68	0.45 0.20, 0.64	0.34 -0.21, 0.61	0.60 0.35, 0.77	0.43 0.18, 0.63	0.19 -0.08, 0.45	0.54 NA, 0.73		0.64 0.44, 0.79	0.59 0.39, 0.74	0.45 0.22, 0.65	0.36 0.14, 0.56	0.54 0.28, 0.74	0.22 -0.05, 0.46	0.39 0.11, 0.62	0.42 0.14, 0.63	0.12 -0.17, 0.39	0.28 -0.01, 0.53	0.33 0.04, 0.57	0.22 - 0.10, 0.49

**11**	0.94 0.88, 0.97	0.91 0.84, 0.96	0.58 0.35, 0.75	0.49 0.24, 0.68	0.64 0.42, 0.79	0.64 0.40, 0.79	0.48 0.24, 0.67	0.55 0.32, 0.73	0.71 0.51, 0.84)	0.72 0.55, 0.83		0.66 0.48, 0.79	0.58 0.36, 0.75	0.48 0.28, 0.64	0.68 0.49, 0.81	0.33 0.07, 0.56	0.55 0.30, 0.72	0.39 0.11, 0.62	0.22 -0.08, 0.49	0.39 0.11, 0.62	0.51 0.26, 0.70	0.41 0.14, 0.63
**12**	0.73 0.57, 0.85	0.73 0.58, 0.85	0.66 0.48, 0.79	0.33 0.09, 0.54	0.50 0.28, 0.68	0.51 0.29, 0.69	0.50 0.28, 0.67	0.43 0.19, 0.63	0.61 0.41, 0.76	0.68 0.50, 0.82	0.72 0.57, 0.83		0.89 0.79, 0.94	0.91 0.85, 0.94	0.64 0.43, 0.78	0.21 -0.04, 0.44	0.48 0.20, 0.69	0.54 0.28, 0.73	0.30 0.03, 0.54	0.60 0.38, 0.76	0.55 0.31, 0.72	0.27 -0.01, 0.52
**13**	0.63 0.45, 0.78	0.64 0.47, 0.78	0.55 0.35, 0.70	0.28 0.04, 0.50	0.47 0.24, 0.65	0.43 0.20, 0.62	0.36 0.12, 0.56	0.34 0.11, 0.55	0.45 0.22, 0.64	0.58 0.40, 0.73	0.60 0.42, 0.73	0.87 0.81, 0.91		0.90 0.83, 0.94	0.52 0.24, 0.72	0.15 -0.11, 0.40	0.49 0.21, 0.68	0.48 0.23, 0.68	0.30 0.02, 0.54	0.59 0.37, 0.75	0.56 0.33, 0.73	0.39 0.11, 0.61
**14**	0.87 0.76, 0.93	0.88 0.77, 0.94	0.63 0.43, 0.78	0.59 0.38, 0.75	0.65 0.44, 0.79	0.61 0.38, 0.77	0.55 0.34, 0.72	0.44 0.18, 0.64	0.77 0.61 0.87	0.79 0.65, 0.88	0.83 0.71, 0.90	0.91 0.84, 0.95	0.83 0.71, 0.91		0.47 0.25, 0.64	0.22 -0.03, 0.45	0.46 0.21, 0.66	0.47 0.24, 0.65	0.43 0.18, 0.63	0.57 0.36, 0.72	0.46 0.23, 0.65	0.34 0.06, 0.57
**15**	0.81 0.71, 0.88	0.82 0.73, 0.89	0.53 0.32, 0.70	0.49 0.27, 0.66	0.63 0.44, 0.77	0.70 0.52, 0.82	0.52 0.31, 0.69	0.45 0.23, 0.64	0.55 0.33, 0.71	0.67 0.51, 0.79	0.78 0.67, 0.85	0.70 0.55, 0.80	0.61 0.40, 0.74	0.72 0.58, 0.83		-0.02 -0.27, 0.24	0.43 0.15, 0.65	0.47 0.19, 0.67	0.34 0.06, 0.57	0.41 0.14, 0.62	0.50 0.25, 0.69	0.34 0.05, 0.57
**16**	0.89 0.80, 0.94	0.82 0.69, 0.91	0.57 0.33, 0.74	0.55 0.31, 0.72	0.59 0.35, 0.76	0.62 0.37, 0.78	0.44 0.17, 0.65	0.37 0.08, 0.60	0.51 0.23, 0.71	0.74 0.56, 0.87	0.88 0.78, 0.93	0.62 0.41, 0.77	0.50 0.29, 0.68	0.81 0.64, 0.91	0.75 0.62, 0.84		0.03 -0.32, 0.28	0.10 -0.37, 0.20	0.05 -0.30, 0.23	0.18 -0.10, 0.44	0.15 -0.14, 0.41	0.09 -0.18, 0.36
**17**	0.65 0.43, 0.81	0.62 0.39, 0.77	0.45 0.18, 0.66	0.45 0.19, 0.65	0.43 0.13, 0.66	0.64 0.42, 0.79	0.34 0.06, 0.57	0.27 -0.01, 0.53	0.44 0.16, 0.66	0.58 0.35, 0.75	0.58 0.36, 0.75	0.50 0.24, 0.71	0.19 -0.25, 0.45	0.53 0.28, 0.72	0.53 0.32, 0.70	0.52 0.24, 0.72		0.28 -0.02, 0.54	0.10 -0.18, 0.38	0.41 0.13, 0.63	0.71 0.51, 0.83	0.46 0.19, 0.67
**18**	0.62 0.40, 0.78	0.53 0.29, 0.71	0.34 0.08, 0.56	0.29 0.02, 0.52	0.13 -0.15, 0.40	0.44 0.17, 0.65	0.23 -0.03, 0.47	0.03 -0.24, 0.30	0.24 -0.04, 0.49	0.70 0.51, 0.82	0.50 0.27, 0.68	0.50 0.24, 0.70	0.50 0.28, 0.67	0.50 0.27, 0.68	0.41 0.04, 0.61	0.55 0.28, 0.73	0.59 0.37, 0.76		0.14 -0.15, 0.42	0.27 -0.01, 0.52	0.36 0.07, 0.59	0.41 0.13, 0.63
**19**	0.58 0.32, 0.78	0.43 0.13, 0.65	0.38 0.10, 0.61	0.33 0.04, 0.57	0.21 -0.10, 0.49	0.26 -0.05, 0.53	0.13 -0.15, 0.40	0.19 -0.11, 0.45	0.36 0.05, 0.60	0.25 -0.06, 0.52	0.40 0.09, 0.64	0.42 0.14, 0.64	0.60 0.38, 0.76	0.53 0.27, 0.73	0.38 0.12, 0.59	0.41 0.11, 0.64	0.35 0.06, 0.59	0.34 0.07, 0.57		0.17 -0.10, 0.43	0.16 -0.13, 0.43	0.10 -0.19, 0.38
**20**	0.51 0.31, 0.67	0.45 0.23, 0.63	0.21 -0.10, 0.45	0.25 0.00, 0.47	0.25 -0.01, 0.48	0.30 0.04, 0.52	0.21 -0.04, 0.44)	0.28 0.03, 0.50	0.22 -0.04, 0.46	0.45 0.23, 0.63	0.49 0.28, 0.66	0.50 0.30, 0.67	0.43 0.22, 0.61	0.49 0.29, 0.66	0.52 0.33, 0.67	0.57 0.38, 0.72	0.39 0.15, 0.58	0.47 0.26, 0.64	0.28 0.03, 0.50		0.50 0.25, 0.69	0.19 -0.10, 0.46
**21**	0.77 0.61, 0.87	0.51 0.25, 0.70)	0.34 0.06, 0.57	0.38 0.11, 0.60	0.22 -0.08, 0.48	0.41 0.13, 0.63	0.16 -0.12, 0.42	0.18 -0.11, 0.45	0.34 0.05, 0.58	0.46 0.20, 0.67	0.69 0.50, 0.82	0.46 0.21, 0.66	0.36 0.11, 0.57	0.53 0.29, 0.72	0.54 0.32, 0.71	0.71 0.52, 0.83	0.48 0.24, 0.68	0.53 0.31, 0.70	0.47 0.22, 0.67	0.46 0.23, 0.64		0.65 0.44, 0.79
**22**	0.67 0.46, 0.81	0.46 0.19, 0.68	0.28 -0.01, 0.53	0.17 -0.12, 0.43	0.09 -0.21, 0.38	0.22 -0.08, 0.50	0.09 -0.19, 0.37	0.09 -0.19, 0.38	0.26 -0.04, 0.52	0.33 0.01, 0.60	0.62 0.40, 0.78	0.45 0.19, 0.65	0.34 0.08, 0.56	0.42 0.13, 0.66	0.46 0.21, 0.65	0.59 0.36, 0.76	0.41 0.14, 0.63	0.53 0.29, 0.72	0.37 0.10, 0.60	0.40 0.16, 0.60	0.83 0.72, 0.90	

Note: 1=weight, 2=bmi, 3=tricepsSF, 4=bicepsSF, 5=subscapularSF, 6=suprailiacSF, 7=calfSF, 8=frontthighSF, 9=abdominalSF, 10=waistC, 11=hipC, 12=upperarmC, 13=upper armflexedC, 14=forearmC, 15=thighC, 16=calfC, 17=biacromialD, 18=bicristalD, 19=humerusD, 20=femurD, 21=height, 22=sitting height (SF=skinfold; C=circumference; D=diameter)
